# RIM and MUNC13 membrane–binding domains are essential for neuropeptide secretion

**DOI:** 10.1083/jcb.202409196

**Published:** 2025-05-12

**Authors:** Fiona H. Murphy, Adlin Abramian, Remco V. Klaassen, Frank Koopmans, Claudia M. Persoon, August B. Smit, Ruud F. Toonen, Matthijs Verhage

**Affiliations:** 1 Department of Human Genetics, Center for Neurogenomics and Cognitive Research (CNCR), Amsterdam University Medical Center (UMC), Amsterdam, Netherlands; 2 Department of Functional Genomics, Vrije Universiteit (VU) Amsterdam, Amsterdam, Netherlands; 3 Department of Molecular and Cellular Neurobiology, Center for Neurogenomics and Cognitive Research (CNCR), Vrije Universiteit (VU) Amsterdam, Amsterdam, Netherlands

## Abstract

Neurons release neurotransmitters from synaptic vesicles (SVs) and neuropeptides from dense-core vesicles (DCVs). The presynaptic proteins RIM and MUNC13 play key roles in both pathways. It remains unclear how DCVs are targeted to release sites and whether RIM and MUNC13 are involved in this process. Here, we show that three membrane-binding domains in RIM and MUNC13 regulate DCV exocytosis differently from SV exocytosis. Using neuropeptide secretion assays with single-vesicle resolution and peptidomics analysis of endogenous neuropeptide release in MUNC13/RIM null neurons, we demonstrate that MUNC13 is essential for DCV exocytosis. The RIM N terminus prevents MUNC13 degradation via the proteasome, and inhibiting proteasomal degradation partially rescues DCV exocytosis in RIM’s absence. Unlike SV exocytosis, the PIP_2_-binding RIM C2B domain and MUNC13 C1-C2B polybasic face are redundant for DCV exocytosis, while the lipid-binding MUNC13 C2C domain is crucial. These results show that RIM and MUNC13 synergistically regulate DCV exocytosis through membrane interactions and reveal new mechanistic differences between SV and DCV exocytosis.

## Teaser

This study examines how RIM and MUNC13 synergistically regulate DCV exocytosis. It demonstrates that MUNC13 is essential for DCV secretion, while RIM prevents its degradation. Key membrane-binding domains of RIM and MUNC13 play differing roles, revealing distinct mechanisms from synaptic vesicle release.

## Introduction

Dense-core vesicles (DCVs) are secretory organelles that store, transport, and secrete neuromodulators (e.g., neuropeptides, neurotrophic factors, and guidance cues) (van den Pol, 2012; Gondré-Lewis et al., 2012). Neuropeptides regulate many complex processes such as circadian rhythm, emotions, and metabolism, and dysregulation of neuropeptide secretion is associated with several clinical phenotypes (van der Klaauw, 2018; Zheng et al., 2021; Chen et al., 2019). In contrast to synaptic vesicles (SVs), DCVs are not recycled at the active zone but are produced in the ER/Golgi and trafficked throughout dendrites and axons to their release sites, which are in and outside of synapses (Persoon et al., 2018). Up to now, studies have shown that the protein machinery that controls DCV exocytosis is largely similar to SV exocytosis: both SV and DCV exocytosis depend on SNARE complex formation involving MUNC13, MUNC18, syntaxin-1, SNAP25, and VAMP2/synaptobrevin (Puntman et al., 2021; Arora et al., 2017; Hoogstraaten et al., 2020; van de Bospoort et al., 2012; Schoch et al., 2001; Shimojo et al., 2015). Previously, we have shown that the active zone protein RIM, and particularly its interaction with MUNC13, is essential for DCV exocytosis (Persoon et al., 2019). It is currently unknown to what extent RIM and MUNC13 perform independent roles in DCV exocytosis and whether their function differs from SV exocytosis. Additionally, it is unclear how DCVs are physically linked to their release sites and whether RIM and MUNC13 play a role in this process.

DCV exocytosis is restored in neurons lacking RIM1/2 by expressing a RIM N-terminal fragment (Persoon et al., 2019) that interacts with RAB3 and MUNC13 (Kaeser et al., 2011). The RIM zinc finger, within the N terminus, binds to the MUNC13 C2A domain, thereby disrupting MUNC13 homodimerization. RIM-MUNC13 heterodimerization promotes SV docking and priming (Deng et al., 2011; Camacho et al., 2017). Multiple studies have reported that MUNC13 protein levels are depleted in RIM1/2 DKO cells (Zarebidaki et al., 2020; Persoon et al., 2019; Deng et al., 2011; Fernández-Busnadiego et al., 2013). Interestingly, in contrast to SV priming, which is only rescued in RIM1/2-deficient neurons by expressing monomeric MUNC13 mutants, DCV exocytosis is also rescued by WT MUNC13 (Deng et al., 2011; Persoon et al., 2019). These observations indicate that MUNC13 compensates for RIM’s function in DCV exocytosis if highly expressed and that its function may differ between SV and DCV exocytosis.

Recent studies showed that RIM and MUNC13 target SVs to the plasma membrane, thereby enabling SV exocytosis (Camacho et al., 2021; Quade et al., 2019; de Jong et al., 2018; Papantoniou et al., 2023). A single mutation in the MUNC13-1 C1-C2B polybasic face (K603E) and a double point mutation in the PIP_2_ phospholipid-binding RIM C2B domain (K1513E/K1515E, referred to as 2E) both ablate binding to the plasma membrane and impair SV exocytosis (Camacho et al., 2021; de Jong et al., 2018). These studies show that the C2B domain of RIM and C1-C2B polybasic face of MUNC13 play a vital, independent role in SV exocytosis. By disrupting the membrane-binding MUNC13 C2C domain, neurotransmitter release is strongly reduced, suggesting a direct stabilizing interaction between MUNC13 and SVs (Quade et al., 2019; Padmanarayana et al., 2021). It is currently unknown whether membrane-binding domains play a role in the targeting and stabilization of DCVs at release sites in and outside of the synapse.

In this study, we aimed to clarify the distinct roles of RIM and MUNC13 and test the importance of their membrane-binding domains in DCV exocytosis. Live-cell imaging at single-vesicle resolution, alongside secretome analysis of endogenous neuropeptides, revealed that DCV exocytosis requires MUNC13, and overexpression of RIM is not sufficient to support DCV exocytosis in the absence of MUNC13. Moreover, MUNC13 levels are severely diminished in the absence of RIM but can be restored by expressing the RIM N terminus or inhibiting the proteasome. Proteasome inhibition also increased DCV exocytosis in RIM DKO neurons. Interestingly, in contrast to SV exocytosis, the C2B domains of RIM and C1-C2B polybasic face of MUNC13 are redundant for DCV exocytosis, while a double point mutation in the MUNC13 C2C domain abolishes DCV exocytosis. These findings suggest that the RIM N terminus is essential for stabilizing MUNC13 and preventing its degradation, enabling DCV exocytosis through interactions at the plasma and vesicle membrane.

## Results

### RIM1 N terminus only rescues DCV exocytosis in the presence of MUNC13

**Figure 1.**
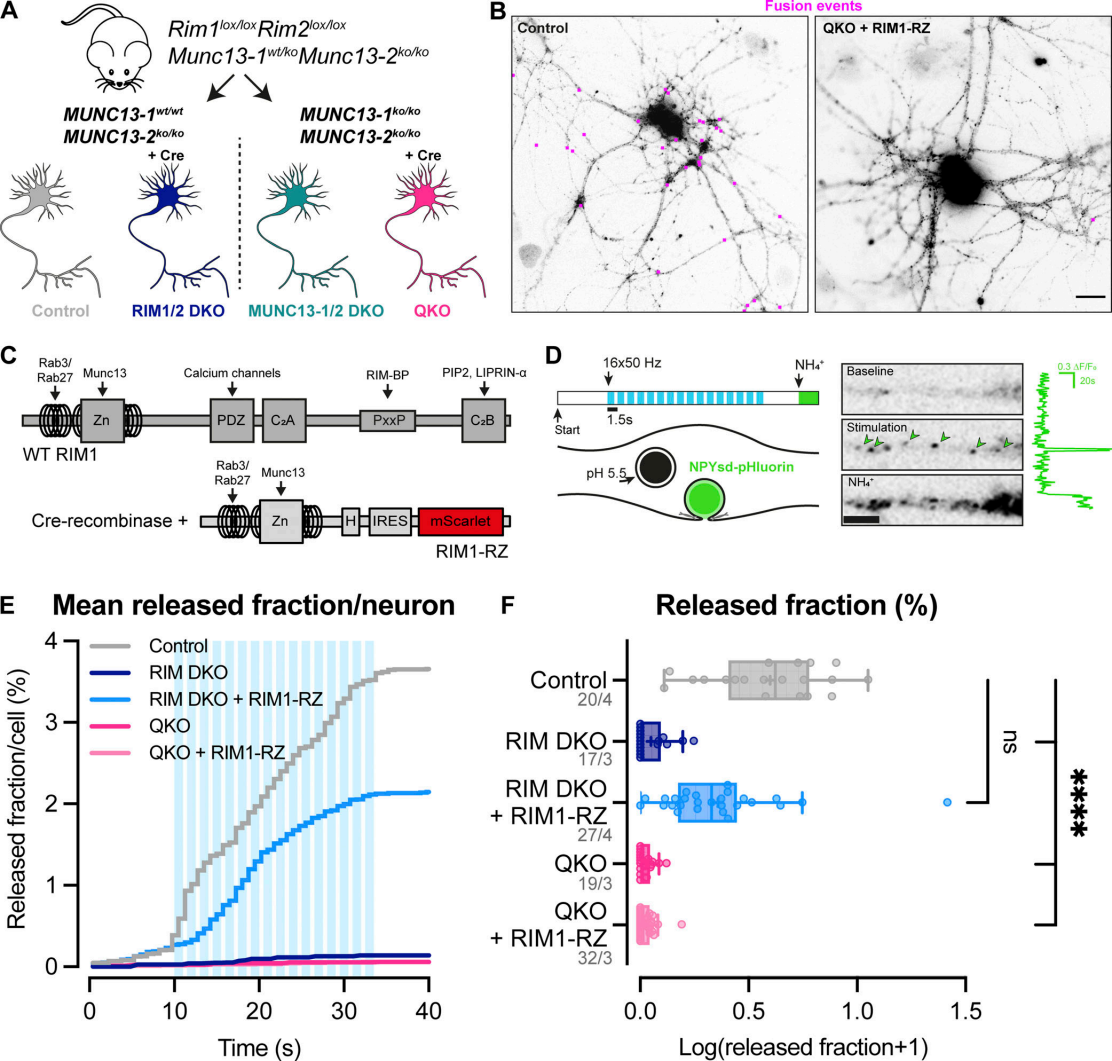
**N-terminal RIM rescues DCV exocytosis in the presence of MUNC13. (A)** Schematic depiction of the RIM1/2 MUNC13-1/2 mouse line used in this study. Control neurons were MUNC13-1 WT and MUNC13-2 KO (see Fig. S1). RIM DKO neurons were generated from MUNC13-1 WT mice upon infection with Cre-recombinase at DIV1. QKO neurons were generated from MUNC13-1 KO mice in the same manner. **(B)** Representative images of single-cultured hippocampal neurons from control (left) and QKO (right) mice. Both images taken during perfusion with NH_4_^+^ buffer. Pink rectangles represent recorded fusion events during repetitive electrical stimulation. Scale bar = 20 μm. **(C)** Schematic illustration of WT full-length RIM1 (top) and the RIM1-RZ N terminus rescue construct (bottom). Key domains and interactions are indicated. Zn: zinc finger domain with surrounding helical regions; H: HA tag; IRES: internal ribosome entry site; mScarlet: mScarlet fluorophore for visualization of expression. Rescue constructs were infected on DIV1. **(D)** Schematic representation of DCV exocytosis assay (WT neurons used as an example). Blue bars represent repetitive electrical stimulation (16 trains of 50 action potentials at 50 Hz). Green bar represents perfusion with 50 mM NH_4_Cl in Tyrode’s buffer. NPYsd-pHluorin is quenched in acidic DCV lumen. Upon exocytosis, a rapid increase in fluorescence is measured due to fluorophore de-quenching. This is followed by a decrease in fluorescence caused by cargo release and/or DCV closing and re-acidifying. Panels right: NPYsd-pHluorin signal during baseline recording, stimulation with fusion events indicated by arrowheads, and perfusion with NH_4_^+^ buffer, showing all DCVs. Scale bar = 10 μm. Green trace shows intensity trace of fusion event indicated by green arrowhead. Neurons were infected with NPYsd-pHluorin on DIV9/10, and DCV exocytosis was recorded on DIV14–16. **(E and F)** DCV exocytosis analysis of control (grey), RIM DKO (dark blue), and QKO (pink) neurons with (light blue and light pink) and without the RIM1-RZ rescue construct. MUNC13-2 KO neurons (MUNC13-1 WT; MUNC13-2 KO) neurons infected with inactive EGFP-tagged Cre-recombinase were used as control. RIM DKO neurons were generated using the same neurons infected with EGFP-tagged Cre-recombinase. QKO neurons were MUNC13 DKO; RIM DKO infected with EGFP-tagged Cre-recombinase. **(E)** Cumulative plot of mean released fraction of DCVs per cell. Released fraction was calculated by dividing the total amount of fusion events by the remaining pool size of DCVs (see Materials and methods for more details). **(F)** Boxplot with Tukey whiskers showing the Log_10_ of released fraction of DCVs per condition. Horizontal line indicates median, and cross indicates mean. *n*/*N* represents number of single neuron observations (*n*) and independent experiments (different nests) (*N*). Individual neurons are represented as dots. Kruskal–Wallis with Dunn’s correction: ns, P = 0.9879. ****P < 0.0001. Additional statistical comparisons not depicted in figure: RIM DKO versus RIM DKO + RIM1-RZ and RIM DKO + RIM1-RZ versus QKO + RIM1-RZ: ***P < 0.0001. RIM DKO versus QKO, RIM DKO + RIM1-RZ versus QKO + RIM1-RZ, and QKO versus QKO + RIM1-RZ: ns, P > 0.9999.

**Figure S1.**
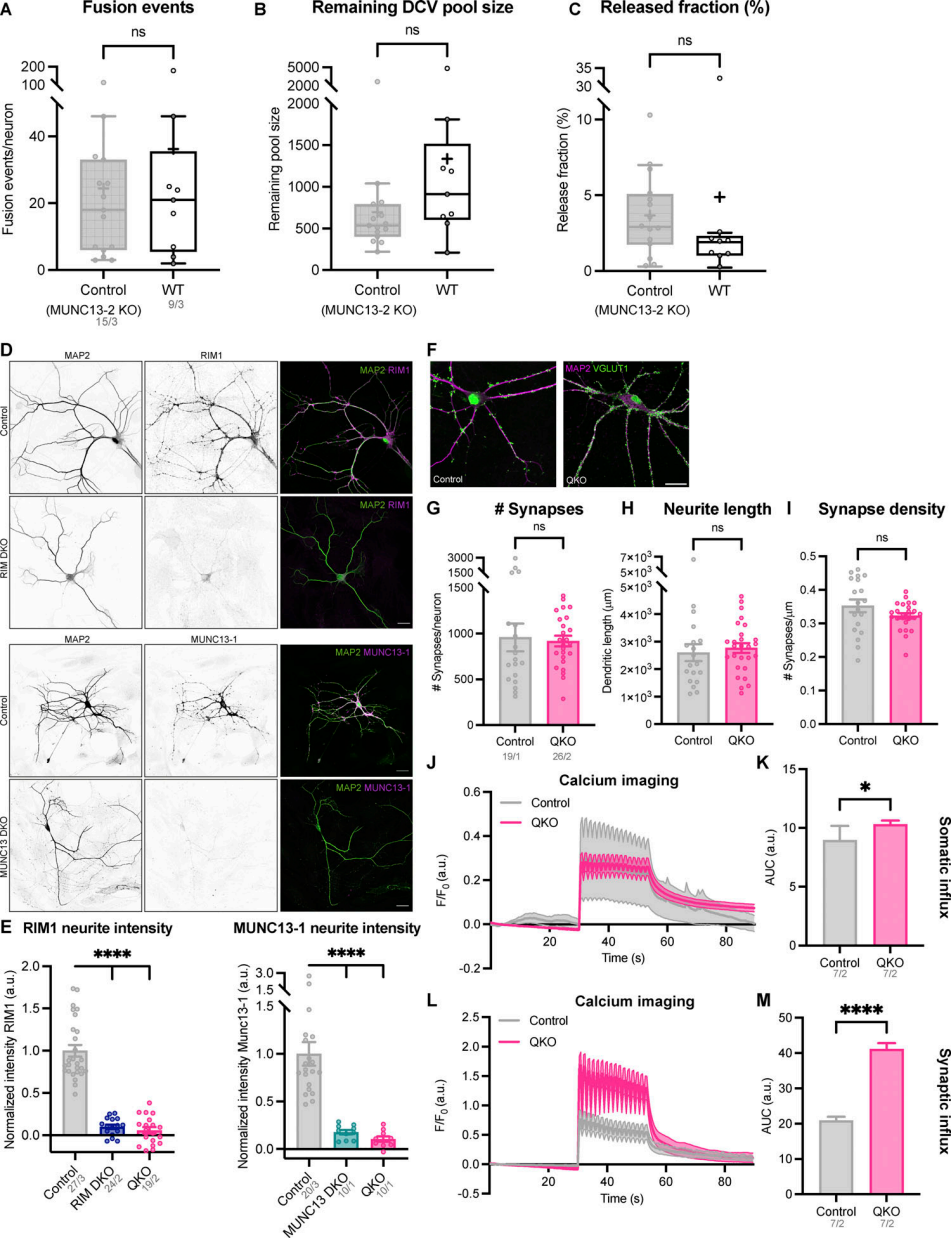
**Validation of MUNC13/RIM mouse line. (A–C)** DCV exocytosis analysis of control (grey) and WT (white) neurons. MUNC13-2 KO neurons (MUNC13-1 WT; MUNC13-2 KO infected with inactive EGFP-tagged Cre-recombinase) were used as control. Boxplot with Tukey whiskers showing (A) number of DCV fusion events per condition, (B) remaining DCV pool size, and (C) released fraction of DCVs. Horizontal line indicates median, and cross indicates mean. *n*/*N* represents number of single neuron observations (*n*) and independent experiments (*N*) (all graphs the same). Individual neurons are represented as dots. Mann–Whitney test: ns (A), P = 0.9417. ns (B), P = 0.1383. ns (C), P = 0.0552. **(D)** Representative confocal images of a single-isolated hippocampal neurons (DIV14) stained for MAP2 (left), RIM1 (top middle) and MUNC13 (bottom middle), in control, RIM DKO, MUNC13DKO, and QKO neurons. **(E)** Mean intensity of RIM (left) or MUNC13 (right) in neurites. Signal intensity is normalized to control. *n*/*N* represents number of single neuron observations (*n*) and independent experiments (*N*). One-way ANOVA with Dunn’s correction: ****P < 0.0001. **(F)** Representative composite confocal image of a single hippocampal neuron (control [top] and QKO [bottom]), immunostained for MAP2 (magenta) and VGLUT1 (green). **(G–I)** Quantification of morphological characteristics. For details on analysis, see Materials and methods. **(G)** Quantification of number of synapses per neuron. Mann–Whitney test: ns, P = 0.3717. **(H)** Average MAP2-positive neurite length per neuron. Mann–Whitney test: ns, P = 0.2874. **(I)** Number of synapses per neurite length (synapse density) per neuron. Mann–Whitney test: ns, P = 0.1430. *n*/*N* represents number of single neuron observations (*n*) and independent experiments (*N*). **(J)** ΔF/F_0_ trace of rise in somatic intracellular calcium (Fluo5-AM) upon repetitive electrical stimulation in control (MUNC13-2 KO) and QKO neurons. Traces were corrected for baseline (first 15 frames) and normalized (for more details, see Materials and methods). **(K)** Area under the curve (AUC) of calcium traces shown in J. *n*/*N* represents number of single neuron observations (*n*) and independent experiments (*N*). *t* test with Welch’s correction: *P = 0.0045. **(L)** ΔF/F_0_ trace of rise in intracellular calcium (Fluo5-AM) in synapses upon repetitive electrical stimulation in control (MUNC13-2 KO) and QKO neurons. Neurons were infected on DIV10 with synapsin-ECFP for synapse identification. Synapses were selected using a mask generated by manually thresholding synapsin-ECFP signal. Soma signal was excluded. Traces were corrected for baseline (first 15 frames) and normalized (for more details, see Materials and methods). **(M)** Area under the curve (AUC) of calcium traces shown in L. *n*/*N* represents number of single neuron observations (*n*) and independent experiments (*N*). *t* test with Welch’s correction: ****P < 0.0001.

**Figure S2.**
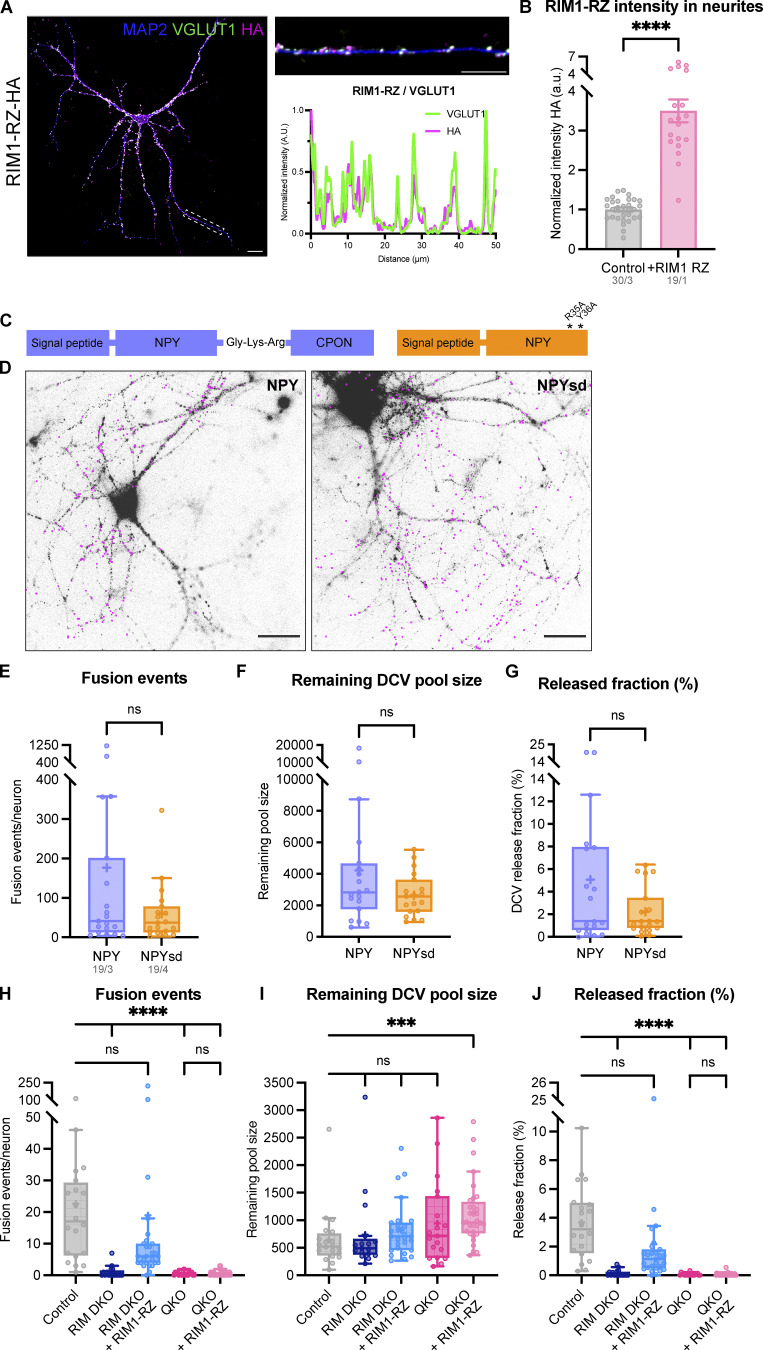
**RIM1-RZ rescue construct expression, DCV exocytosis kinetics using NPYsd-pHluorin reporter, and extra exocytosis parameters belonging to** Fig. 1 **. (A)** Left: Representative composite confocal image of a single hippocampal neuron (DIV14) expressing RIM1-RZ–HA, immunostained for MAP2 (blue), VGLUT1 (green), and HA (magenta). Right: Example zoom of (A). Line plots show normalized fluorescence intensity across displayed neurite. **(B)** Mean intensity of RIM1-RZ–HA in neurites depicted in A. Signal was normalized to control levels. *t* test: ****P < 0.0001. *n*/*N* represents number of single neuron observations (*n*) and independent experiments (*N*). **(C)** Schematic representation of NPY (left) and NPYsd (right), with asterisks representing mutations in NPY receptor–binding residues. **(D)** Representative images of single-cultured hippocampal neurons from control neurons infected with NPY-pHluorin (left) and NPYsd-pHluorin (right) (left). Both images taken during perfusion with NH_4_^+^ buffer. Pink rectangles represent recorded fusion events during repetitive electrical stimulation. Scale bar = 20 μm. **(E–G)** DCV exocytosis analysis of control neurons infected with NPY-pHluorin (purple) and NPYsd-pHluorin (orange). Boxplot with Tukey whiskers showing (E) number of DCV fusion events per condition, (F) remaining DCV pool size, and (G) released fraction of DCVs. Horizontal line indicates median, and cross indicates mean. *n*/*N* represents number of single neuron observations (*n*) and independent experiments (*N*) (all graphs the same). Individual neurons are represented as dots. Mann–Whitney test: ns (E), P = 0.4054. ns (F), P = 0.4879. ns (G), P = 0.4181. **(H–J)** DCV exocytosis analysis of control (grey), RIM DKO (dark blue), and QKO (pink) neurons with (light blue and light pink) and without the RIM1-RZ rescue construct. MUNC13-2 KO neurons (MUNC13-1 WT; MUNC13-2 KO neurons infected with inactive EGFP-tagged Cre-recombinase) were used as control. RIM DKO neurons were generated using the same neurons infected with EGFP-tagged Cre-recombinase. QKO neurons were MUNC13 DKO infected with EGFP-tagged Cre-recombinase. Boxplot with Tukey whiskers showing (H) total number of fusion events per neuron, (I) remaining DCV pool, and (G) release fraction of DCVs per condition. Horizontal line indicates median, and cross indicates mean. *n*/*N* represents number of single neuron observations (*n*) and independent experiments (*N*) (same as Fig. 1). Individual neurons are represented as dots. Kruskal–Wallis with Dunn’s correction: ns (A, control versus RIM DKO + RIM1-RZ), P > 0.9999. ns (A, QKO versus QKO + RIM1-RZ), P > 0.9999. ns (B, control versus RIM DKO), P > 0.9999. ns (B, control versus RIM DKO + RIM1-RZ), P = 0.5436. ns (B, control versus QKO), P = 0.5661. ns (C, control versus RIM DKO + RIM1-RZ), P = 0.9879. ns (C, QKO versus QKO + RIM1-RZ), P > 0.9999. ****P < 0.0001. ***P = 0.0007.

Previously, we showed that the RIM1 N terminus, which interacts with RAB3 and MUNC13, is sufficient to drive DCV exocytosis in neurons lacking all RIM1 (α and β) and RIM2 (α, β, and γ) isoforms (Persoon et al., 2019). However, it is unknown whether MUNC13 is an essential component in this process. To test this, we generated a mouse line lacking all RIM and MUNC13 isoforms. Deletion of all MUNC13 isoforms (MUNC13-1 and MUNC13-2), similar to the deletion of all RIM isoforms, is lethal. To generate RIM1/2; MUNC13-1/2 quadruple null mutant (QKO) mice, we crossbred *Rim1/2*^*lox/lox*^ (Kaeser et al., 2011) and *Munc13-1*^*wt/ko*^*/Munc13-2*^*ko/ko*^ (Varoqueaux et al., 2002) mice, which result in either *Munc13-1*^*wt/wt*^ or *Munc13-1*^*ko/ko*^ offspring (Fig. 1 A). *Munc13-2*^*ko/ko*^ was used to increase the chances of the correct genotypes within the same nest. The loss of MUNC13-2 does not affect DCV exocytosis when compared with WT neurons (non-littermates) (Fig. S1, A–C). Active (or inactive) Cre-recombinase was added at DIV1 to *Munc13-1*^*wt/wt*^ to generate control and RIM1/2 cDKO (referred to as RIM DKO) neurons, and to *Munc13-1*^*ko/ko*^ to generate MUNC13-1/2 DKO (referred to as MUNC13 DKO) and RIM1/2; MUNC13-1/2 QKO (referred to as QKO) neurons (Fig. 1 A; and Fig. S1, D and E). QKO neurons did not display obvious deficits in neurite length or number of synapses, and synaptic calcium influx was not impaired. In fact, total calcium influx was increased in QKO neurons in both somatic and synaptic regions (Fig. S1, F–M). To assess DCV exocytosis, we used single-isolated hippocampal neurons grown on glia micro-islands (Fig. 1 B). RIM DKO and QKO neurons were infected with a RIM1 N terminus rescue construct (RIM1-RZ) (Deng et al., 2011), which localized to presynaptic regions (Fig. 1 C; and Fig. S2, A and B). While the presynaptic localization of this and following rescue constructs in this manuscript shows they are intact and accurately targeted to the synapse, it does not fully exclude incomplete targeting to the DCV environment, as DCVs are also localized outside of the synapse. DCV fusion events were detected at single-vesicle resolution using a signaling-dead neuropeptide-Y (NPYsd; Fig. S2 C) variant tagged with pH-sensitive GFP (pHluorin). NPYsd-pHluorin was previously shown to localize exclusively to DCVs in primary neurons (Nassal et al., 2022; Subkhangulova et al., 2023). The fusion kinetics of this reporter were similar to WT NPY-pHluorin (Fig. S2, D–G). DCV fusion was triggered using electrical field stimulation of 16 bursts of 50 action potentials at 50 Hz (Fig. 1 D, left). Upon fusion pore opening, the vesicle lumen rapidly deacidifies, causing a sudden increase in fluorescence of the NPY-pHluorin reporter (Fig. 1 D, right). We recorded DCV exocytosis in all experiments on DIV14–16. Upon stimulation, RIM DKO and QKO neurons showed almost no DCV exocytosis (Fig. 1, E and F; and Fig. S2, H–J). Expression of RIM1-RZ rescued DCV exocytosis in the RIM DKO, as we observed before (Persoon et al., 2019). However, no rescue was observed in QKO neurons (Fig. 1, E and F). The total number of DCVs (pool size) was similar in all experimental groups (Fig. S2 I). Hence, the RIM N terminus requires the presence of MUNC13 to restore DCV exocytosis in the absence of full-length RIM.

### MUNC13 is essential for DCV exocytosis

**Figure 2.**
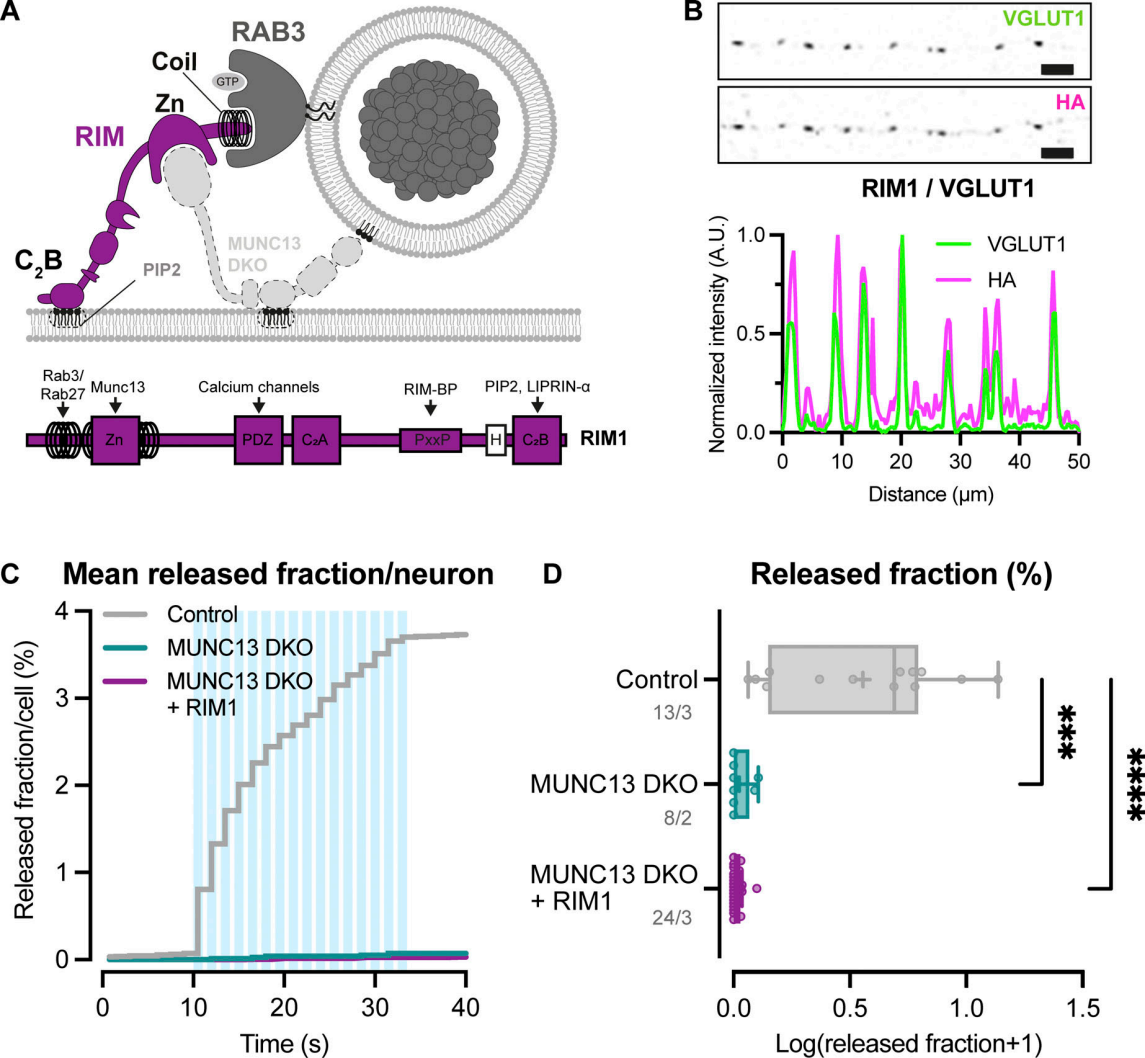
**Loss of MUNC13 is not compensated by re-expression of RIM. (A)** Schematic depiction of RIM interacting with a DCV at the plasma membrane in the absence of MUNC13. Below: Domain structures of RIM1. Key domains and interactions are indicated. H: HA-tag for localization studies. Rescue construct was infected on DIV1. **(B)** Example zoom of QKO neuron-expressing HA-tagged RIM1-WT rescue construct. Neurites were labeled with dendritic marker MAP2 (blue) (shown in Fig. S3), presynaptic marker VGLUT1 (green), and RIM1 was visualized with anti-HA (magenta). Line plots show normalized fluorescence intensity across displayed neurite. Scale bars = 5 μm. **(C and D)** DCV exocytosis analysis of control neurons (grey), MUNC13 DKO (teal), and MUNC13 DKO neurons rescued with RIM1 (dark purple). **(C)** Cumulative plot of mean released fraction of DCVs per cell. **(D)** Boxplot with Tukey whiskers showing release fraction of DCVs per condition. Horizontal line indicates median, and cross indicates mean. *n*/*N* represents number of single neuron observations (*n*) and independent experiments (*N*). Individual neurons are represented as dots. Kruskal–Wallis test with Dunn’s correction: ***P = 0.0004. ****P < 0.0001. Additional statistical comparison not depicted in figure: MUNC13 DKO versus MUNC13 DKO + RIM1: ns, P > 0.9999.

**Figure S3.**
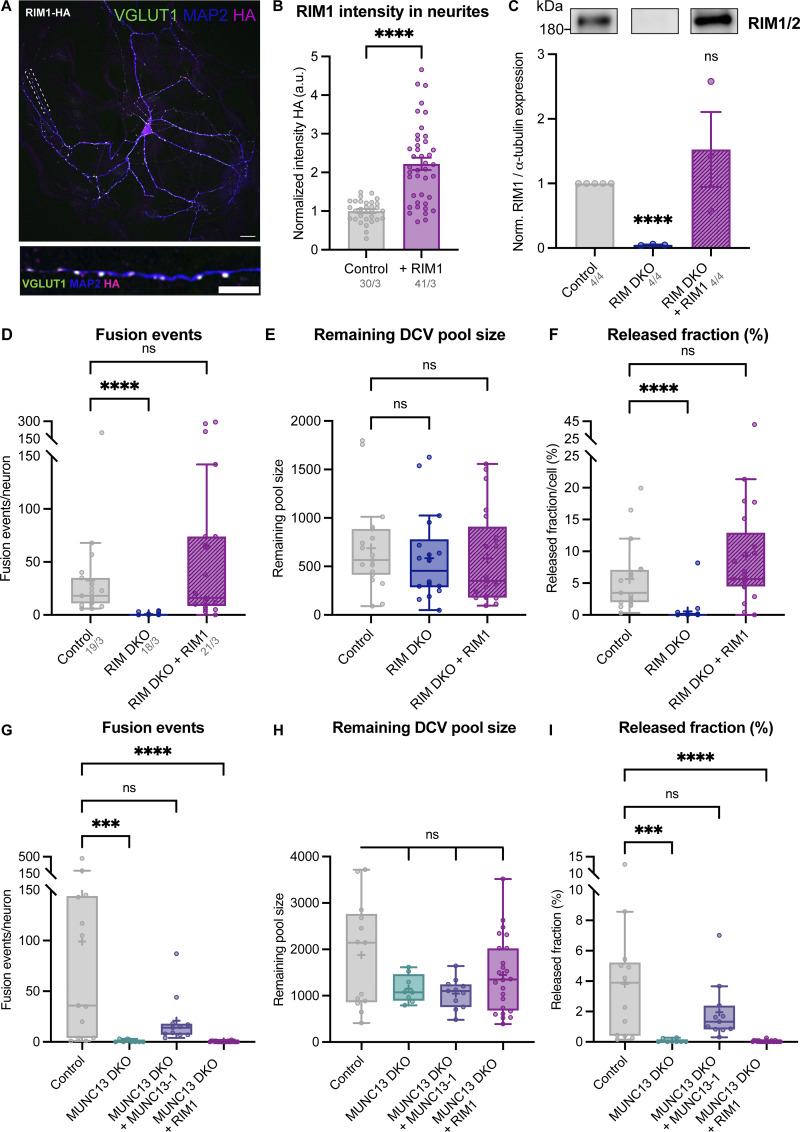
**RIM1 rescue construct validation and extra exocytosis parameters belonging to** Fig. 2 **. (A)** Representative composite confocal image of a single hippocampal neuron (DIV14) expressing RIM1-HA, immunostained for MAP2 (blue), VGLUT1 (green), and HA (magenta). **(B)** Mean intensity of RIM1-HA in neurites depicted in A. Signal was normalized to control levels. Mann–Whitney test: ****P < 0.0001. *n*/*N* represents number of single neuron observations (*n*) and independent experiments (*N*). **(C)** Top: Immunoblot labeled for RIM1 (left) in control, RIM DKO, and RIM DKO infected with RIM1-HA (whole cell lysates). Bottom: Mean intensity of RIM immunoblot signal. Signal was first normalized to α-tubulin signal from the same lane and then normalized to the control signal. One sample *t* test: ****P < 0.0001. ns, P = 0.4608. **(D–F)** DCV exocytosis analysis of control (grey) RIM DKO (dark blue) and RIM DKO rescued with RIM1-HA (purple) neurons. Boxplot with Tukey whiskers showing (D) total fusion events per neurons, (E) total remaining DCV pool, and (F) release fraction of DCVs per condition. Horizontal line indicates median, and cross indicates mean. *n*/*N* in D represents number of single neuron observations (*n*) and independent experiments (*N*) (same for D–F). Individual neurons are represented as dots. Kruskal–Wallis test with Dunn’s correction: ****P < 0.0001. ns (A), P >0.9999. ns (B, control versus RIM DKO), P = 0.7383. ns (B, control versus RIM DKO + RIM1), P = 0.5280. ns (C), P = 0.7233. **(G–I)** DCV exocytosis analysis of control (grey), MUNC13 DKO (teal) with MUNC13-1 rescue construct (blue), and RIM1 rescue construct (dark purple). MUNC13-2 KO neurons (MUNC13-1 WT; MUNC13-2 KO) were used as control. Boxplot with Tukey whiskers showing (G) total number of fusion events per neuron, (H) remaining DCV pool, and (I) release fraction of DCVs per condition. Horizontal line indicates median, and cross indicates mean. *n*/*N* represents number of single neuron observations (*n*) and independent experiments (*N*) (same as Fig. 2). Individual neurons are represented as dots. Kruskal–Wallis test with Dunn’s correction: ns (A), P > 0.9999. ***P = 0.0002. ****P < 0.0001. ns (B, control versus MUNC13 DKO), P = 0.8495. ns (B, control versus MUNC13 DKO + MUNC13-1), P = 0.3183. ns (B, control versus MUNC13 DKO + RIM1), P > 0.9999. ns (C), P > 0.9999. Source data are available for this figure: SourceData FS3.

**Figure S4.**
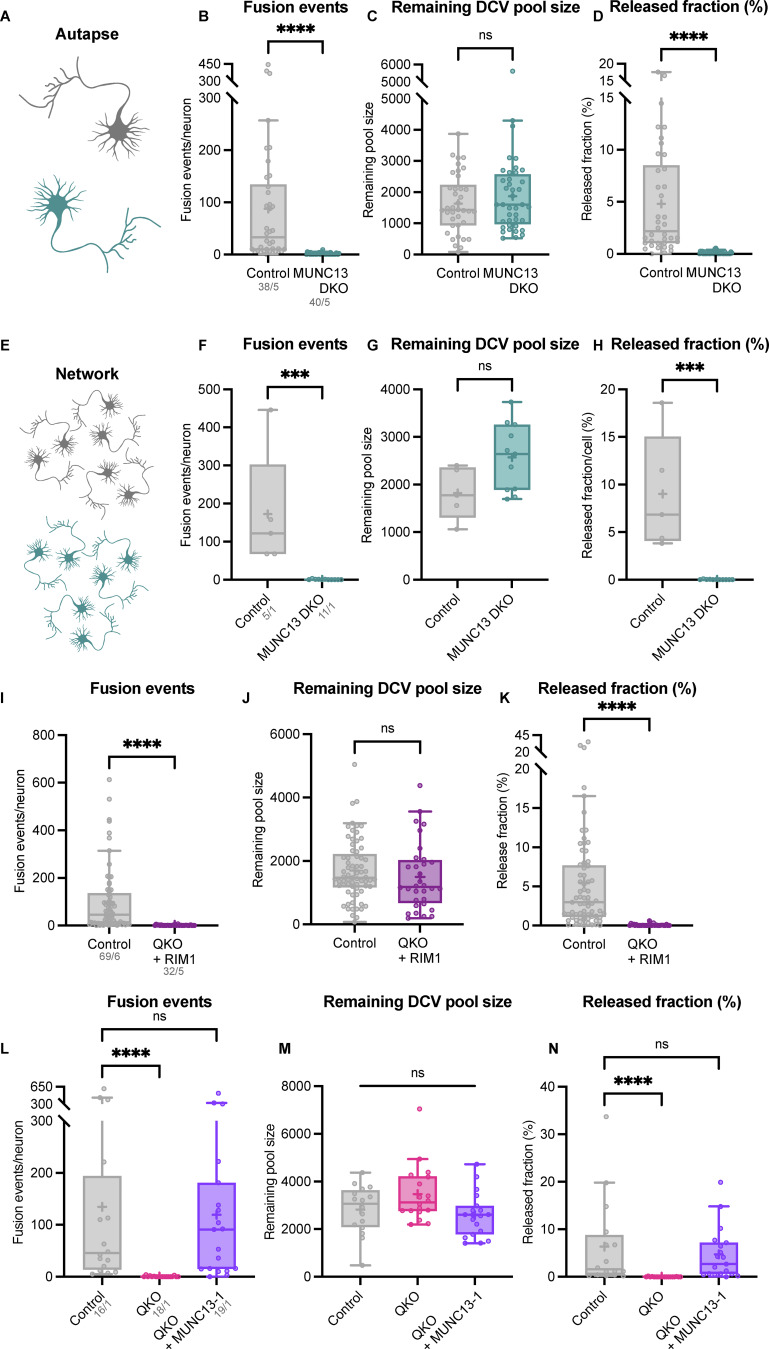
**MUNC13 DKO in autaptic and network neuronal cultures; MUNC13, but not RIM, rescues DCV exocytosis in QKO neurons. (A–D)** DCV exocytosis analysis of autaptic control (grey) and MUNC13 DKO (teal) neurons. **(A)** Experimental setup. Boxplot with Tukey whiskers showing (B) total fusion events per neurons, (C) total remaining DCV pool, and (D) release fraction of DCVs per condition. Horizontal line indicates median, and cross indicates mean. *n*/*N* represents number of single neuron observations (*n*) and independent experiments (*N*). Individual neurons are represented as dots. Mann–Whitney test: ****P < 0.0001. ns, P = 0.4661. **(E–H)** DCV exocytosis analysis of network cultures of control (grey) and MUNC13 DKO (teal) neurons. **(E)** Experimental setup. Boxplot with Tukey whiskers showing (F) total fusion events per neurons, (G) total remaining DCV pool, and (H) release fraction of DCVs per condition. Horizontal line indicates median, and cross indicates mean. *n*/*N* represents number of single neuron observations (*n*) and independent experiments (*N*). Individual neurons are represented as dots. Mann–Whitney test: ***P = 0.0002. ns, P = 0.0687. **(I–K)** DCV exocytosis analysis of control (grey) and QKO rescued with RIM1-HA (purple) neurons. MUNC13-2 KO neurons (MUNC13-1 WT; MUNC13-2 KO) were used as control. Boxplot with Tukey whiskers showing (I) total number of fusion events per neuron, (J) remaining DCV pool, and (K) release fraction of DCVs per condition. Horizontal line indicates median, and cross indicates mean. *n*/*N* represents number of single neuron observations (*n*) and independent experiments (*N*). Individual neurons are represented as dots. Mann–Whitney test: ****P < 0.0001. ns, P = 0.1680. **(L–N)** DCV exocytosis of control (grey), QKO (magenta), and QKO rescued with MUNC13-1-FLAG (bright purple) neurons. MUNC13-2 KO neurons (MUNC13-1 WT; MUNC13-2 KO) were used as control. Boxplot with Tukey whiskers showing (L) total number of fusion events per neuron, (M) remaining DCV pool, and (*N*) release fraction of DCVs per condition. Horizontal line indicates median, and cross indicates mean. *n*/*N* represents number of single neuron observations (*n*) and independent experiments (*N*). Individual neurons are represented as dots. Kruskal–Wallis test: ****P < 0.0001. ns (fusion events), P > 0.9999. ns (remaining pool size, control versus QKO) P = 0.3984. ns (remaining pool size, control versus QKO + MUNC13) P = 0.5735. ns (released fraction) P = >0.9999.

Previously, we showed that MUNC13 overexpression rescues DCV exocytosis in RIM DKO neurons, indicating that high cellular levels of MUNC13 compensate for RIM’s function in DCV exocytosis (Persoon et al., 2019). To further investigate the interplay between MUNC13 and RIM, we assessed DCV exocytosis in MUNC13 DKO single neurons and tested whether overexpression of full-length RIM compensates for any potential deficits (Fig. 2 A). HA-tagged RIM1-WT localized to presynaptic regions and resulted in a one to twofold increase in RIM1 levels in RIM DKO neurons compared with control (Fig. 2 B and Fig. S3, A–C). Full-length RIM1-WT, like RIM1-RZ, rescued DCV exocytosis to control levels in RIM DKO neurons (Fig. S3, D–F), as shown before (Persoon et al., 2019). In both autapses and neuronal network cultures, loss of MUNC13 resulted in a ∼98% reduction in DCV exocytosis, while DCV pool sizes remained similar (Fig. 2, C and D; and Fig. S4, A–H). RIM1-WT did not rescue DCV exocytosis in the absence of MUNC13 (Fig. 2, C and D; and Fig. S3, G–I). DCV exocytosis was rescued in MUNC13 DKO when MUNC13-1 is re-expressed (Fig. S3, G–I). Re-expression of RIM1-WT on a QKO background, similar to MUNC13 DKO, did not rescue DCV exocytosis (Fig. S4, I–K). Furthermore, DCV exocytosis was rescued in QKO neurons by re-expressing MUNC13-1, in line with previous findings using RIM DKO neurons (Fig. S4, L–N) (Persoon et al., 2019). Altogether, these data show that MUNC13 is essential for DCV exocytosis and that MUNC13 carries out crucial functions that are not compensated for by RIM.

### Endogenous neuropeptide secretion is blocked upon loss of RIM or MUNC13

**Figure 3.**
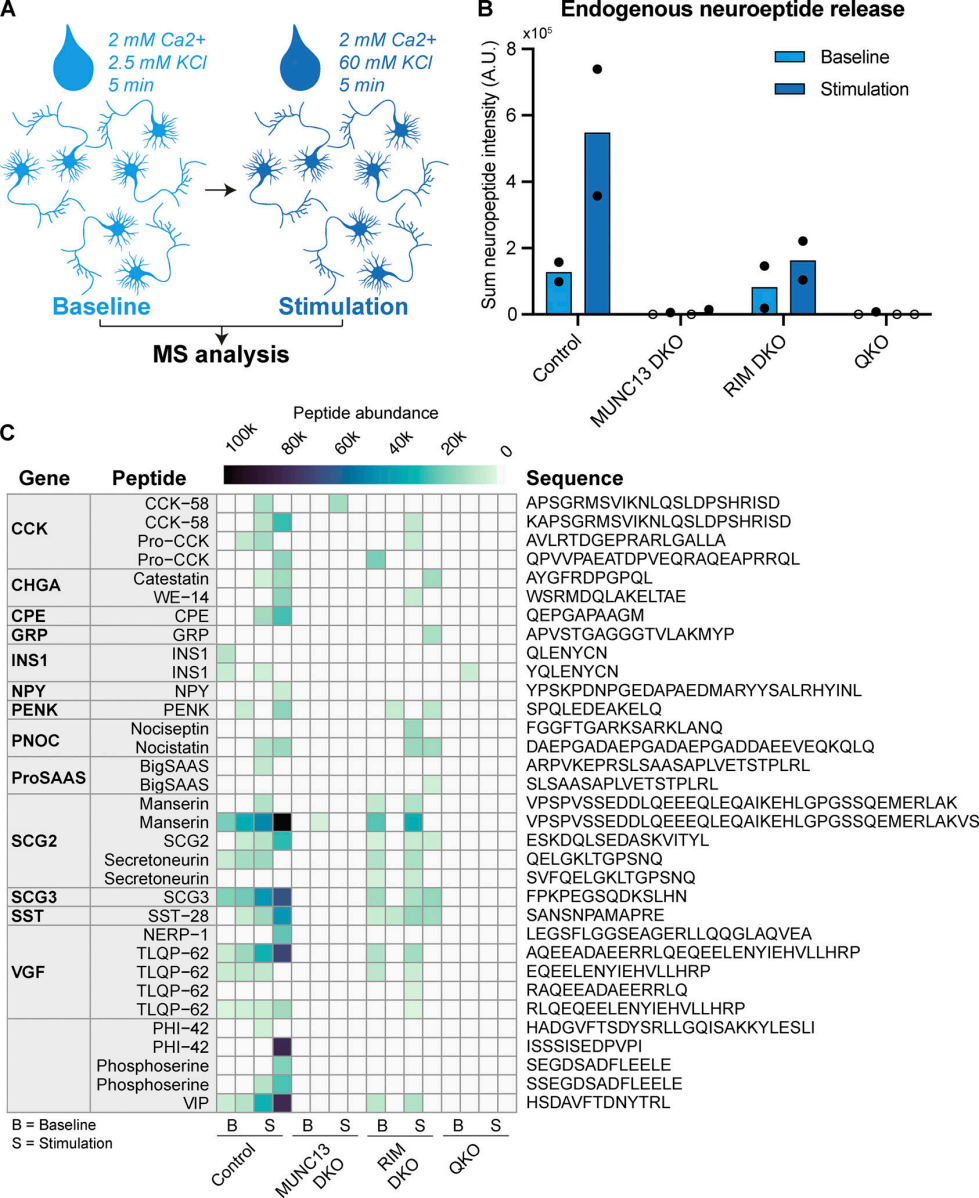
**Loss of RIM and/or MUNC13 blocks endogenous neuropeptide release. (A)** Illustration of experimental setup. Neurons were grown for 21 days at high density (30 K/well). Baseline and stimulation solutions were collected after 5 min incubation at RT. **(B)** Sum of measured neuropeptide intensities. Bar plot with technical replicates shown as points (open circles indicate complete absence). **(C)** Heat map depicting peptide abundance values for each neuropeptide across all samples.

**Figure S5.**
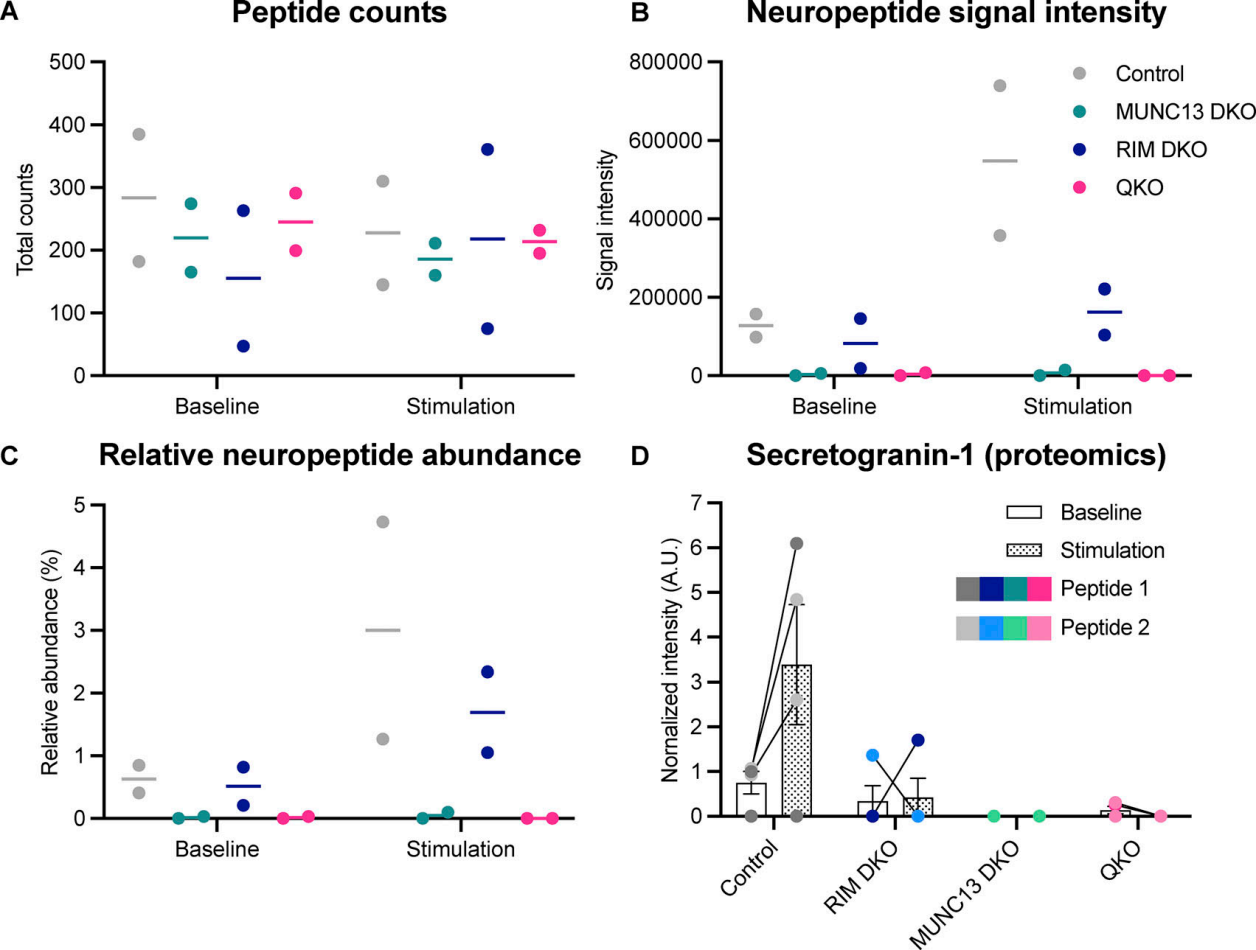
**Peptide counts and abundance and proteomics analysis of SCG-1 belonging to** Fig. 3 **. (A)** Total peptide counts per condition. **(B)** Signal intensity of all measured neuropeptides per condition. **(C)** Relative abundance of neuropeptides per condition, normalized to total abundance. **(D)** SCG-1 peptide intensity measured using LC/MS proteomics (Thanou et al., 2023). Upon stimulation, SCG-1 peptides are only increased in the control.

Most studies on the mechanisms of neuropeptide secretion rely on overexpressed reporter constructs to detect DCV exocytosis. While this approach enables precise detection of fusion events in real-time with single-vesicle resolution, it does not directly assess endogenous neuropeptide release. Previously, we showed that using an ELISA assay to measure endogenous BDNF secretion is a feasible method to validate findings (Persoon et al., 2019). However, this method is limited to measuring one neuropeptide at a time. To determine whether the loss of RIM and MUNC13 affects endogenous neuropeptide release, we analyzed the secretome of neurons lacking RIM and/or MUNC13 using mass spectrometry analysis. Network cultures of each genotype (control, RIM DKO, MUNC13 DKO, and QKO) were exposed to a baseline and stimulation medium for 5 min each, after which the media were collected and measured using mass spectrometry (Fig. 3 A). At baseline, endogenous neuropeptide release was similar between control and RIM DKO neurons but was nearly fully abolished in MUNC13 DKO and QKO neurons (Fig. 3 B). Upon stimulation, neuropeptide secretion was significantly reduced in RIM DKO and completely absent in MUNC13 DKO and QKO neurons (Fig. 3 B and Fig. S5). Analysis at the level of individual peptides further highlights these differences (Fig. 3 C). While some peptides (PNOC, SCG2, and SST derivatives) remained partially secreted in RIM DKO neurons, others (CCK, CPE, and PY derivatives) were completely absent (Fig. 3 C). Incomplete infection efficiency with Cre virus may contribute to differences in exocytosis inhibition. In conclusion, endogenous neuropeptide release is significantly impaired in RIM DKO neurons and nearly abolished in MUNC13 DKO and QKO neurons, underscoring the critical roles of these proteins in neuropeptide release.

### N-terminal RIM protects MUNC13 from degradation

**Figure 4.**
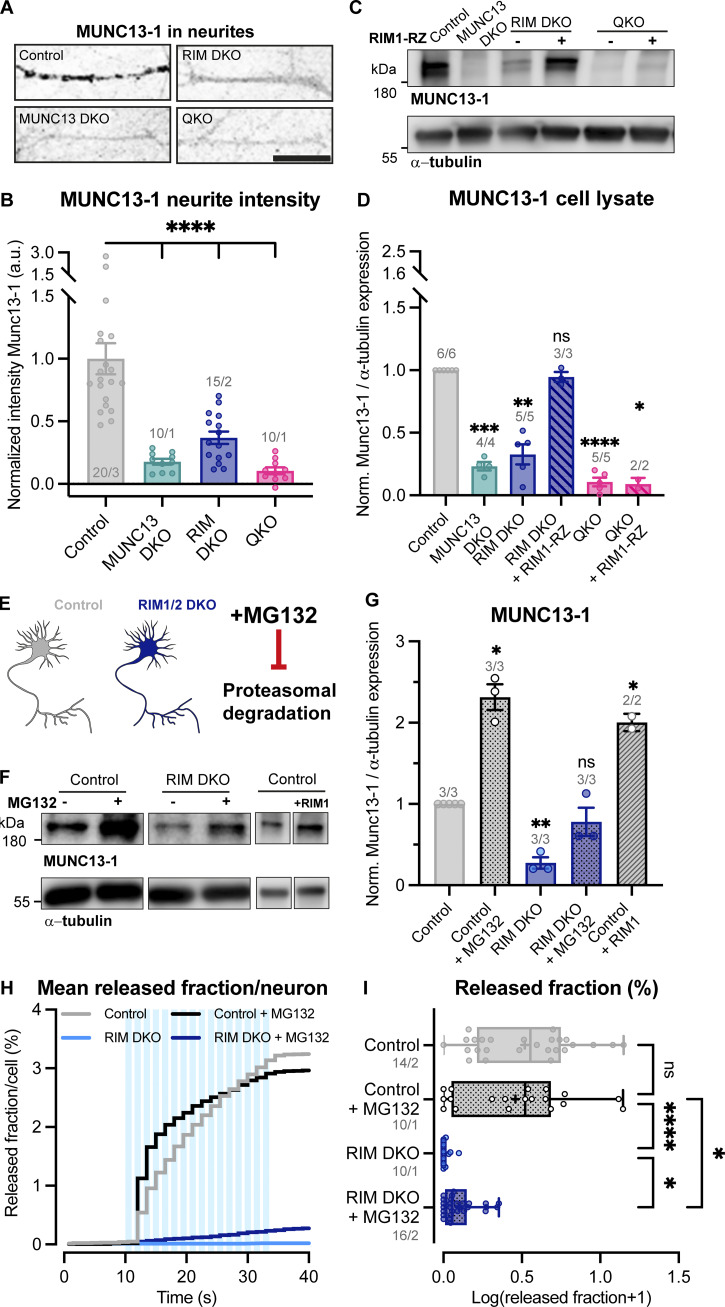
**Loss of RIM increases proteasomal degradation of MUNC13. (A)** Example zoom of control, MUNC13 DKO, RIM DKO, and QKO neurites labeled with MUNC13-1. Scale bar = 20 μm. **(B)** Mean intensity of MUNC13-1 in neurites depicted in A. Signal was normalized to control levels. One-way ANOVA with Dunnett’s correction: ****P < 0.0001. *n*/*N* represents number of single neuron observations (*n*) and independent experiments (*N*). **(C)** Immunoblot labeled for MUNC13-1 in control, MUNC13 DKO and RIM DKO, and QKO with and without RIM1-RZ whole cell lysates. Blots were probed for α-tubulin (bottom) to control for protein loading. **(D)** Mean intensity of MUNC13-1 immunoblot signal shown in C. Signal was first normalized to α-tubulin signal from the same lane and then normalized to the control signal. *n*/*N* represents number of single neuron observations (*n*) and independent experiments (*N*). One sample *t* test: ***P = 0.0002. **P = 0.0011. ns, P = 0.3027. ****P < 0.0001. *P = 0.0246. **(E)** Illustration of the experimental workflow. Control and RIM DKO neurons were used to test the effect of proteasome inhibition on MUNC13-1 levels and DCV exocytosis. **(F)** Immunoblot labeled for MUNC13-1 (top) and α-tubulin (bottom) in control and RIM DKO lysates. 10 μM MG132 was added to control or RIM DKO neurons for 6 h before lysing cells. **(G)** Mean intensity of MUNC13-1 immunoblot signal. Signal was first normalized to α-tubulin signal from the same lane and then normalized to the control signal. *n*/*N* represents number of single neuron observations (*n*) and independent experiments (*N*). One sample *t* test: *P (control + MG132) = 0.0143. *P (RIM DKO) = 0.0145. ns, P = 0.3352. *P (control + RIM1) = 0.0476. **(H and I)** DCV exocytosis analysis of control neurons (grey), control + MG132 (dotted grey), RIM DKO (blue), and RIM DKO + MG132 (dotted dark blue) neurons. **(H)** Cumulative plot of mean released fraction of DCVs per cell. **(I)** Boxplot with Tukey whiskers showing release fraction of DCVs per condition. Horizontal line indicates median, and cross indicates mean. *n*/*N* represents number of single neuron observations (*n*) and independent experiments (*N*). Individual neurons are represented as dots. Kruskal–Wallis test with Dunn’s correction: control versus control + MG132: ns, P > 0.9999. Control + MG132 versus RIM DKO: ****P < 0.0001. Control + MG132 versus RIM DKO + MG132: *P = 0.0268. RIM DKO versus RIM DKO + MG132: *P = 0.0469. Additional statistical comparisons not depicted in figure: Control versus RIM DKO: ****P < 0.0001. Control versus RIM DKO + MG132: ***P = 0.0001. Source data are available for this figure: SourceData F4.

**Figure S6.**
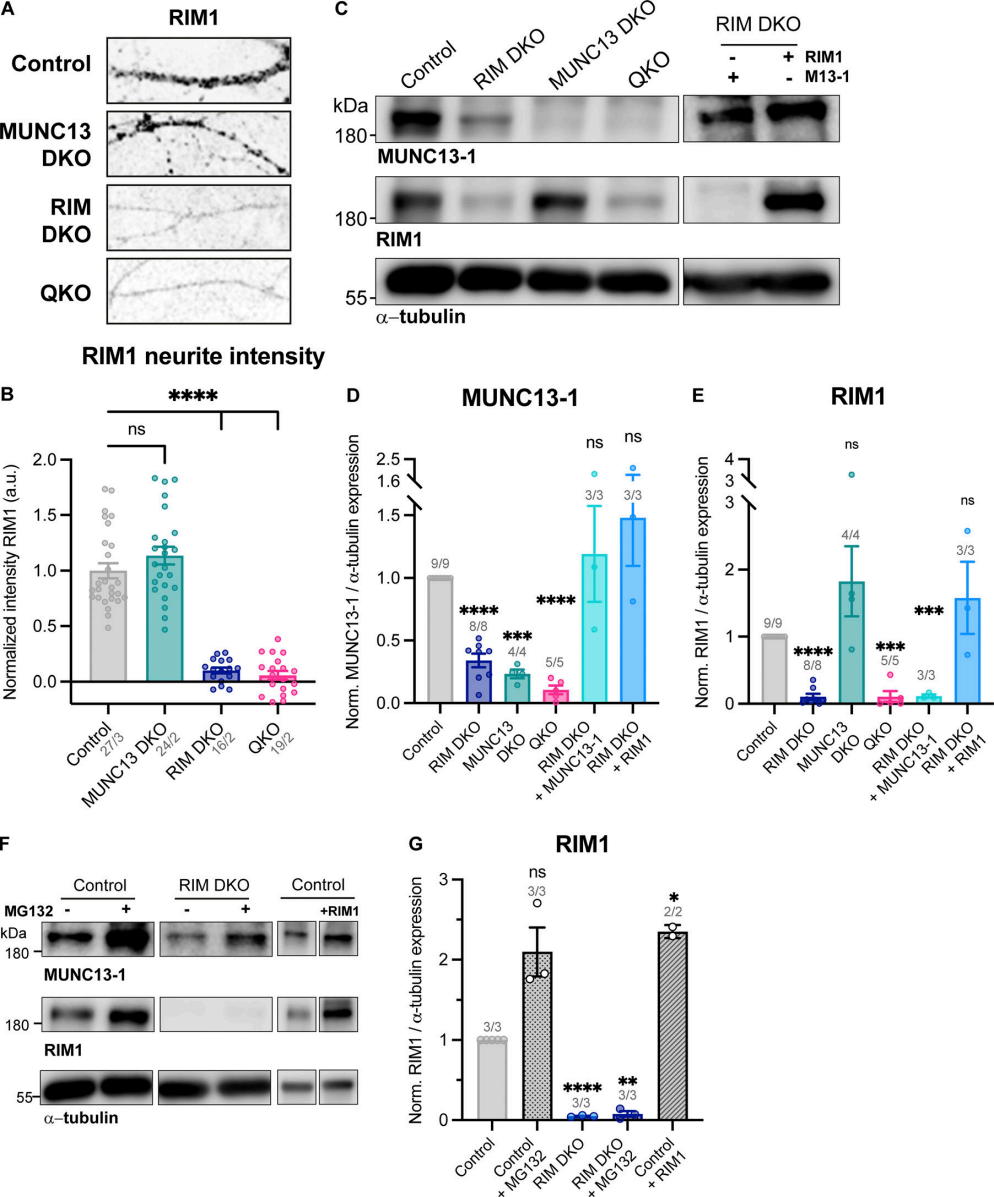
**RIM expression levels are unaffected in MUNC13 DKO. (A)** Example zoom of control, MUNC13 DKO, RIM DKO, and QKO neurites labeled with RIM. Scalebar = 20 μm. **(B)** Mean intensity of RIM1 in neurites depicted in A. Signal was normalized to control levels. One-way ANOVA with Dunnett’s correction: ****P < 0.0001. ns, P = 0.2691. **(C)** Immunoblot labeled for MUNC13-1 (top) and RIM1 (middle) in control, MUNC13 DKO, RIM DKO, and QKO whole cell lysates. In RIM DKO neurons, the RIM1 rescue construct was added at DIV1. **(D)** Mean intensity of MUNC13-1 immunoblot signal. Signal was first normalized to α-tubulin signal from the same lane and then normalized to the control signal. One sample *t* test: ****P < 0.0001. ***P = 0.0002. ns (control versus RIM DKO + RIM1), P = 0.3378. ns (control versus RIM DKO + MUNC13-1), P = 0.6653. **(E)** Mean intensity of RIM1 immunoblot signal. Signal was first normalized to α-tubulin signal from the same lane and then normalized to the control signal. One sample *t* test: ****P < 0.0001. ns, P = 0.2135. ***P (control versus QKO) = 0.0004. ***P (control versus RIM DKO + MUNC13-1) = 0.0008. ns (control versus RIM DKO + RIM1), P = 0.3964. **(F)** Immunoblot labeled for MUNC13-1 (top), RIM1 (middle), and α-tubulin (bottom) in control and RIM DKO lysates. 10 μM MG132 was added to control or RIM DKO neurons for 6 h before lysing cells. **(G)** Mean intensity of RIM1 immunoblot signal. Signal was first normalized to α-tubulin signal from the same lane and then normalized to the control signal. One sample *t* test: ns, P = 0.0688. ****P < 0.0001. **P = 0.0015. Source data are available for this figure: SourceData FS6.

**Figure S7.**
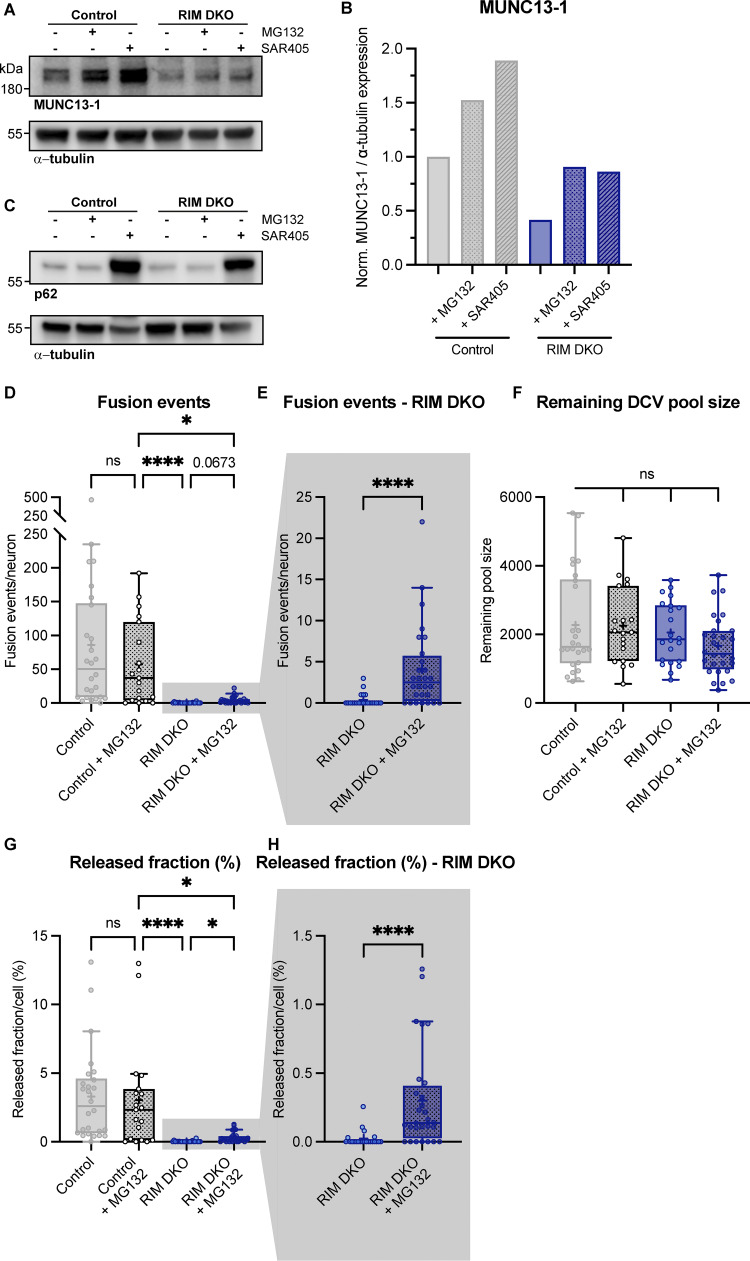
**MUNC13-1 levels increase upon inhibition of autophagy and extra exocytosis parameters belonging to** Fig. 4 **. (A)** Immunoblot labeled for MUNC13-1 (top) and α-tubulin (bottom) in control and RIM DKO lysates. 10 μM MG132 or 5 μM SAR405 was added to control and RIM DKO neurons for 6 or 24 h, respectively. **(B)** Mean intensity of MUNC13-1 signal. Signal was first normalized to α-tubulin signal from the same lane and then normalized to the control signal. *N* = 1. **(C)** Immunoblot labeled for p62 (top) and α-tubulin (bottom). Increase in p62 signal indicates successful inhibition of autophagy. **(D–H)** DCV exocytosis analysis of control neurons (grey), control + MG132 (dotted grey), RIM DKO (blue), and RIM DKO + MG132 (dotted dark blue) neurons. Boxplot with Tukey whiskers showing (D and E) total fusion events per neuron, (F) remaining DCV pool size, and (G and H) release fraction of DCVs per condition. Horizontal line indicates median, and cross indicates mean. *n*/*N* represents number of single neuron observations (*n*) and independent experiments (*N*), same as in Fig. 4. **(D)** Kruskal–Wallis with Dunn’s correction: control versus control + MG132: ns, P > 0.9999. Control + MG132 versus RIM DKO: ****P < 0.0001. Control + MG132 versus RIM DKO + MG132: *P = 0.0158. RIM DKO versus RIM DKO + MG132: ns, P = 0.0673. Additional statistical comparisons not depicted in figure: control versus RIM DKO: ****P < 0.0001. Control versus RIM DKO + MG132: ****P > 0.0001. **(E)** Mann–Whitney test: ****P > 0.0001. **(F)** Kruskal–Wallis with Dunn’s correction: all comparisons P > 0.9999. **(G)** Kruskal–Wallis with Dunn’s correction: control versus control + MG132: ns, P > 0.9999. Control + MG132 versus RIM DKO: ****P < 0.0001. Control + MG132 versus RIM DKO + MG132: *P = 0.0268. RIM DKO versus RIM DKO + MG132: *P = 0.0469. Additional statistical comparisons not depicted in figure: control versus RIM DKO: ****P < 0.0001. Control versus RIM DKO + MG132: ***P = 0.0001. **(H)** Mann–Whitney test: ****P = 0.0001. Source data are available for this figure: SourceData FS7.

The RIM zinc finger domain binds to the MUNC13 C2A domain to form a heterodimer, thereby inhibiting MUNC13 homodimerization (Lu et al., 2006; Deng et al., 2011). As a homodimer, MUNC13 is functionally inactive and tends to aggregate (Lu et al., 2006; Deng et al., 2011; Andrews-Zwilling et al., 2006). Multiple reports showed that loss of RIM results in reduced levels of MUNC13 (Zarebidaki et al., 2020; Persoon et al., 2019; Fernández-Busnadiego et al., 2013), presumably due to nonfunctional MUNC13 being targeted to degradation pathways. These data suggest that RIM acts as a chaperone to MUNC13 by activating its functionality and preventing its degradation. To test this in our model system, we probed protein levels of RIM and MUNC13 in RIM DKO, MUNC13 DKO, and QKO neurons. Semiquantitative immunofluorescence analysis revealed a marked decrease in MUNC13 levels in RIM DKO neurons (Fig. 4, A and B). Western blot analysis confirmed this observation and showed that re-expression of the zinc finger containing RIM1-RZ construct in RIM DKO neurons rescues MUNC13 levels to control (Fig. 4, C and D). In the MUNC13 DKO, RIM levels were not significantly affected (Fig. S6, A–E). These results show that MUNC13 protein levels indeed depend on RIM expression and that the RIM N terminus is sufficient to rescue the reduction in MUNC13 protein levels. To show that the RIM N terminus prevents the degradation of MUNC13, we added the proteasome inhibitor MG132 to control and RIM DKO neurons for 6 h (Fig. 4 E). MUNC13 levels were rescued in RIM DKO cells upon addition of MG132, indicating that at least part of the diminished MUNC13 levels is due to proteasomal degradation, which is normally curbed by MUNC13-RIM heterodimerization (Fig. 4, F and G). Similar results were observed when autophagy was inhibited, indicating that MUNC13 is also targeted to autophagy pathways (Fig. S7, A–C). In control cells, RIM and MUNC13 levels were also increased in the presence of MG132, showing that RIM and MUNC13 are under continuous control by the proteasome (Fig. 4, F and G; and Fig. S6, F and G). To further strengthen the notion that MUNC13 levels depend on RIM, we overexpressed full-length RIM1-WT in control neurons. A strong increase in MUNC13 protein levels was observed (Fig. 4, F and G). Next, we assessed whether proteasome inhibition rescues DCV exocytosis in RIM DKO neurons. A 6-h incubation with MG132 caused a small yet distinct increase in DCV exocytosis in RIM DKO neurons, while DCV pool sizes were unaffected (Fig. 4, H and I; and Fig. S7, D–H). Altogether, our results indicate that RIM affects MUNC13 levels by protecting it from protein degradation and that restoring MUNC13 levels in the absence of RIM is sufficient to partially rescue DCV exocytosis.

### RIM and MUNC13 membrane–binding domains are functionally redundant for DCV exocytosis

**Figure 5.**
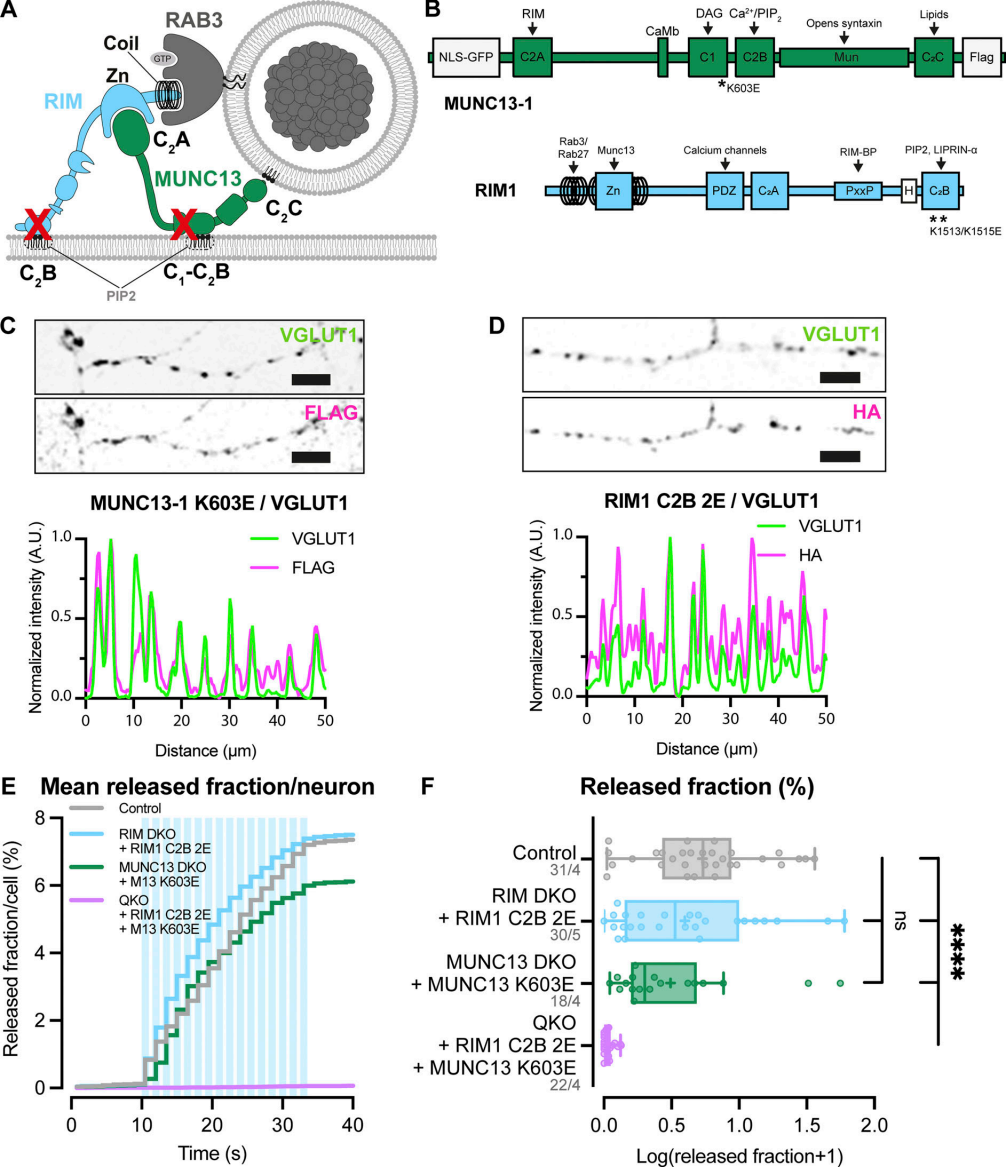
**RIM and MUNC13 membrane–binding domains are redundant for DCV exocytosis. (A)** Schematic depiction of RIM and MUNC13 interactions with a DCV at the plasma membrane. The C2B domains of RIM and C1-C2B polybasic face of MUNC13 both interact with the plasma membrane. **(B)** Domain structures of MUNC13-1 and RIM1 rescue constructs. Key domains and interactions are indicated. NLS-GFP: GFP fluorophore with a nuclear localization signal for visualization of successful infection; FLAG, HA: FLAG- or HA-tag for localization studies. Rescue constructs were infected on DIV1. **(C)** Example zoom of MUNC13 DKO neuron-expressing FLAG-tagged MUNC13-1 K603E rescue construct. Neurites were labeled with dendritic marker MAP2 (blue), presynaptic marker VGlUT1 (green), and MUNC13 K603E was visualized with anti-FLAG (magenta). Line plots show normalized fluorescence intensity across displayed neurite. Scale bars = 5 μm. **(D)** Example zoom of QKO neuron-expressing HA-tagged RIM1 C2B 2E rescue construct. Neurites were labeled with dendritic marker MAP2 (blue) (shown in Fig. S8), presynaptic marker VGlUT1 (green), and RIM1 C2B 2E was visualized with anti-HA (magenta). Line plots show normalized fluorescence intensity across displayed neurite. Scale bars = 5 μm. **(E and F)** DCV exocytosis analysis of control (grey), RIM DKO rescued with RIM1 C2B 2E (light blue), MUNC13 DKO rescued with MUNC13 K603E (green), and QKO rescued with RIM1 C2B 2E and MUNC13 K603E (purple) neurons. **(E)** Cumulative plot of mean released fraction of DCVs per cell. **(F)** Boxplot with Tukey whiskers showing release fraction of DCVs per condition. Horizontal line indicates median, and cross indicates mean. *n*/*N* represents number of single neuron observations (*n*) and independent experiments (*N*). Kruskal–Wallis with Dunn’s correction: ns (control versus RIM DKO + RIM1 C2B 2E), P > 0.9999. ns (control versus MUNC13 DKO + MUNC13 K603E), P = 0.9823. ****P < 0.0001. Additional statistical comparison not depicted in figure: RIM DKO + RIM1 C2B 2E versus MUNC13 DKO + MUNC13 K603E: ns, P > 0.9999.

**Figure S8.**
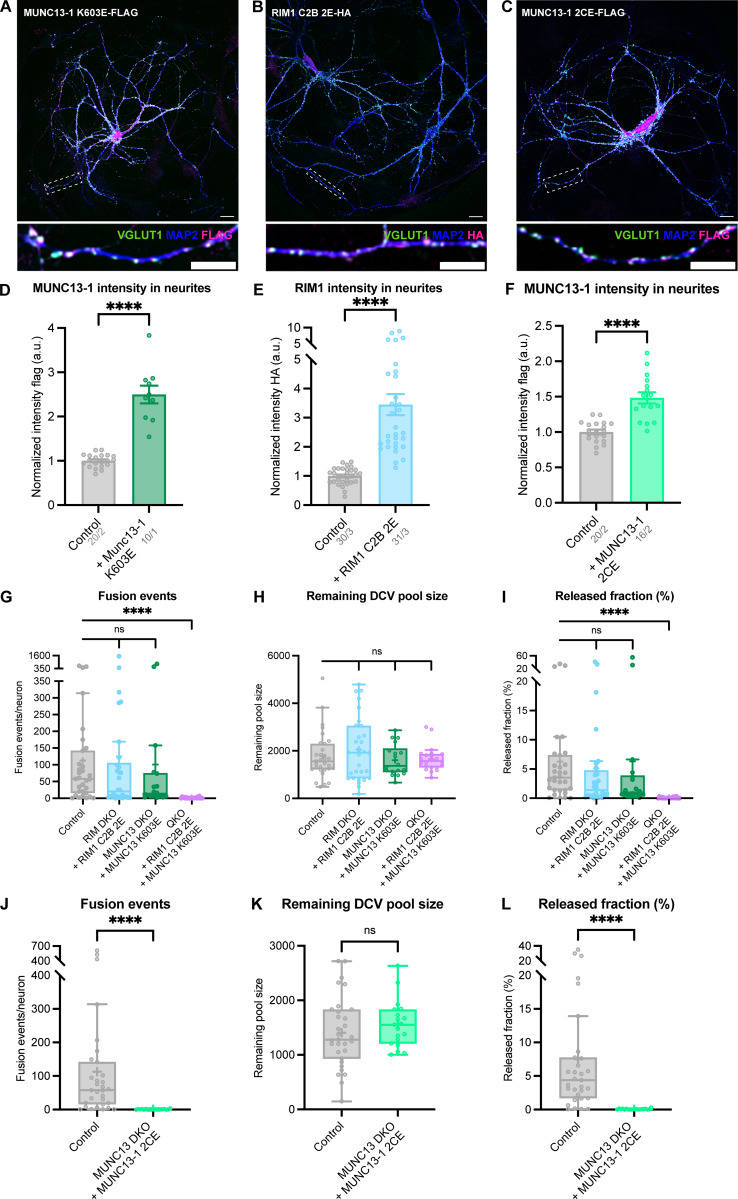
**MUNC13 K603E, RIM1 C2B 2E, and MUNC13 2CE validation and extra exocytosis parameters belonging to** Figs. 5 and 6**. (A–C)** Representative composite confocal image of hippocampal neurons (DIV14) expressing MUNC13 K603E-FLAG (A), RIM C2B 2E-HA, (B) or MUNC13 2CE-FLAG, immunostained for MAP2 (blue), VGLUT1 (green), and HA or FLAG (magenta). **(D)** Mean intensity of MUNC13 K603E in neurites depicted in A. Signal was normalized to control levels. Unpaired *t* test: ****P < 0.0001. *n*/*N* represents number of single neuron observations (*n*) and independent experiments (*N*). **(E)** Mean intensity of RIM1 C2B 2E in neurites depicted in B. Signal was normalized to control levels. Mann–Whitney test: ****P < 0.0001. *n*/*N* represents number of single neuron observations (*n*) and independent experiments (*N*). **(F)** Mean intensity of MUNC13 2CE in neurites depicted in A. Signal was normalized to control levels. *t* test: ****P < 0.0001. *n*/*N* represents number of single neuron observations (*n*) and independent experiments (*N*). **(G–I)** DCV exocytosis analysis of control (grey), RIM DKO rescued with RIM1 C2B 2E (light blue), MUNC13 DKO rescued with MUNC13 K603E (green), and QKO rescued with RIM1 C2B 2E and MUNC13 K603E (purple) neurons. Boxplot with Tukey whiskers showing (G) total fusion events per neuron, (H) remaining DCV pool size, and (I) release fraction of DCVs per condition. Horizontal line indicates median, and cross indicates mean. *n*/*N* represents number of single neuron observations (*n*) and independent experiments (*N*), same as in Fig. 5. **(G)** Kruskal–Wallis with Dunn’s correction: ns (control versus RIM DKO + RIM1 C2B 2E), P = 0.8477. ns (control versus MUNC13 DKO + MUNC13 K603E), P > 0.9999. ****P < 0.0001. **(H)** Kruskal–Wallis with Dunn’s correction: ns (control versus RIM DKO + RIM1 C2B 2E), P = 0.3522. ns (control versus MUNC13 DKO + MUNC13 K603E), P > 0.9999. ns (control versus QKO + RIM1 C2B 2E + MUNC13 K603E), P = 0.3820. **(I)** Kruskal–Wallis with Dunn’s correction: ns (control versus RIM DKO + RIM1 C2B 2E), P = 0.9589. ns (control versus MUNC13 DKO + MUNC13 K603E), P > 0.4911. ****P < 0.0001. **(J–L)** DCV exocytosis analysis of control (grey), MUNC13 DKO rescued with MUNC13 2CE (light green). Boxplot with Tukey whiskers showing (J) total fusion events per neuron, (K) remaining DCV pool size, and (L) release fraction of DCVs per condition. Horizontal line indicates median, and cross indicates mean. *n*/*N* represents number of single neuron observations (*n*) and independent experiments (*N*), same as in Fig. 6. Mann–Whitney test: ****P < 0.0001. ns, P = 0.3548.

RIM and MUNC13 both contain a membrane-binding domain: the PIP_2_-binding C2B domain of RIM and the C1-C2B polybasic region of MUNC13 (Camacho et al., 2021; de Jong et al., 2018) (Fig. 5 A). It was previously shown that mutations in these domains of RIM and MUNC13 disrupt neurotransmitter release (de Jong et al., 2018; Camacho et al., 2021). To study the importance of these interactions for DCV exocytosis, we expressed PIP_2_-binding–deficient HA-tagged RIM1 (K1513E/K1515E, named 2E hereafter) and FLAG-tagged MUNC13-1 (K603E) in RIM DKO, MUNC13 DKO, and QKO neurons (Fig. 5, A and B). Both constructs localized to synaptic regions, as shown by co-localization of presynaptic marker VGLUT1 (Fig. 5, C and D; and Fig. S8, A, B, and D–E). Upon stimulation, RIM1 C2B 2E fully rescued DCV exocytosis in RIM DKO neurons and MUNC13 K603E rescued exocytosis in MUNC13 DKO neurons to >80% of control levels (Fig. 5, E and F). These results show that DCV exocytosis is not blocked by the loss of individual RIM and MUNC13 membrane interactions, meaning that these interactions are dispensable for DCV exocytosis, in stark contrast to SV release. Interestingly, when both RIM C2B 2E and MUNC13 K603E were expressed in QKO neurons, DCV exocytosis was not rescued (Fig. 5, E and F). The remaining pool size was not decreased in any of the conditions (Fig. S8, G–I). These results show that at least one functional membrane-binding domain from MUNC13 or RIM is needed for DCV exocytosis.

### The MUNC13 C2C–lipid interaction is essential for neuropeptide secretion

**Figure 6.**
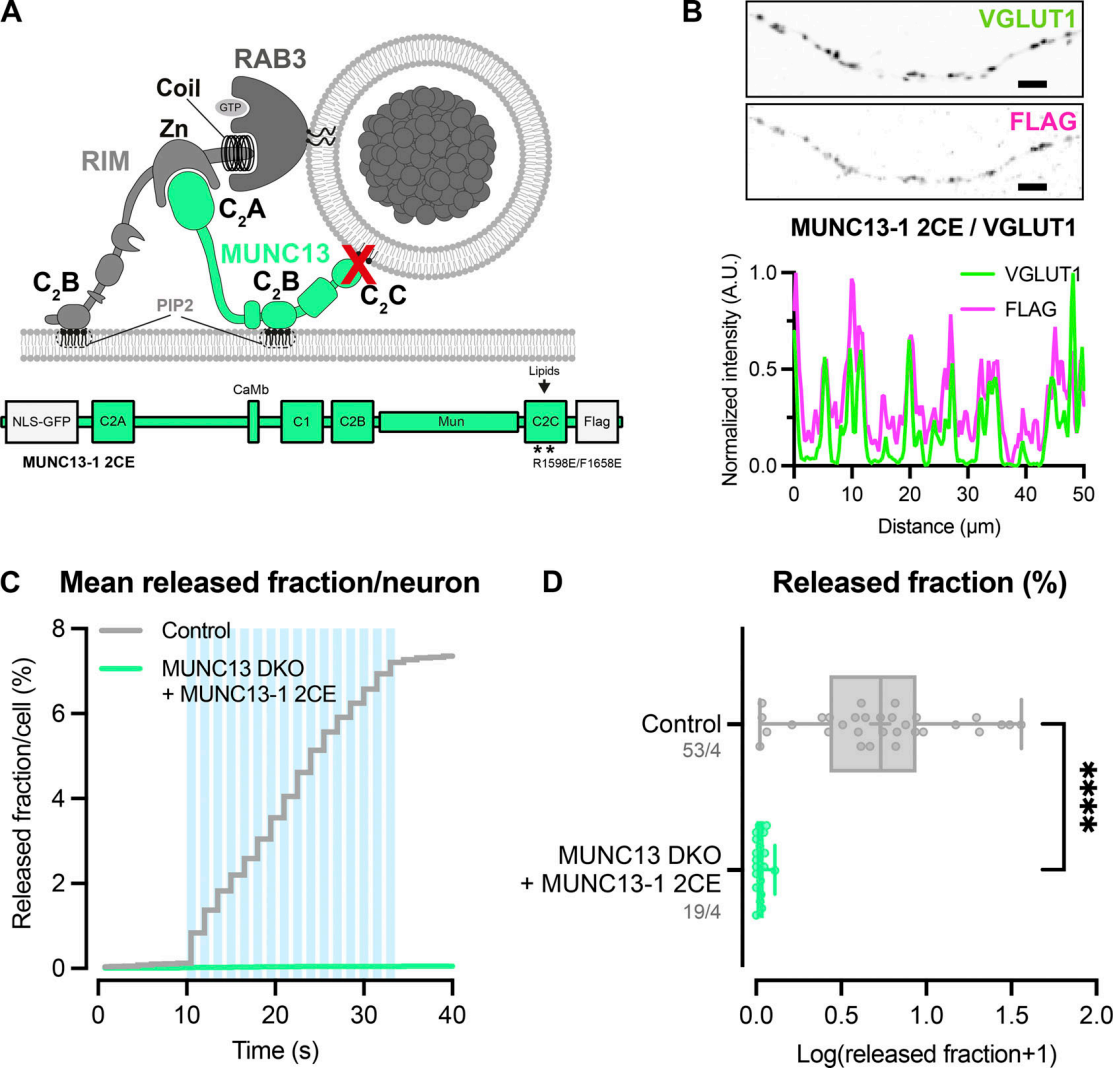
**MUNC13 C2C domain is essential for DCV exocytosis. (A)** Schematic depiction of RIM and MUNC13 interactions with a DCV at the plasma membrane. The C2C domain of MUNC13 interacts with lipids in the vesicle membrane. The α-helical region of RIM interacts with the vesicle through RAB3. Red crosses indicate perturbation of the protein–vesicle interaction. Below: Domain structures of MUNC13-1 and RIM1. Key domains and interactions are indicated. NLS-GFP: GFP fluorophore with a nuclear localization signal for visualization of successful infection; FLAG: FLAG-tag for localization studies. **(B)** Example zoom of MUNC13 DKO neuron-expressing FLAG-tagged MUNC13-1 R1598E/F1658E rescue construct. Neurites were labeled with dendritic marker MAP2 (blue) (shown in Fig. S8), presynaptic marker VGLUT1 (green), and MUNC13 R1598/F1658E (C2C 2E) was visualized with anti-FLAG (magenta). Line plots show normalized fluorescence intensity across displayed neurite. Scale bars = 5 μm. **(C and D)** DCV exocytosis analysis of control (grey) and MUNC13 DKO rescued with MUNC13 2CE (light green). **(C)** Cumulative plot of mean released fraction of DCVs per cell. **(D)** Boxplot with Tukey whiskers showing released fraction of DCVs per condition. Horizontal line indicates median, and cross indicates mean. *n*/*N* represents number of single neuron observations (*n*) and independent experiments (*N*). Individual neurons are represented as dots. Mann–Whitney test: ****P < 0.0001.

In addition to the plasma membrane, MUNC13 and RIM may both also interact with vesicle membranes preceding exocytosis; MUNC13 through its C2C domain (Quade et al., 2019; Padmanarayana et al., 2021), while RIM interacts with vesicle-bound RAB3 through its N-terminal α-helical region (Fig. 6 A). Previously, we showed the relevance of the latter (Persoon et al., 2019). To test the importance of the MUNC13-C2C–vesicle interaction, we expressed a FLAG-tagged MUNC13-1 construct that contains two point mutations in its C2C domain (R1598E/F1658E, hereafter referred to as C2C 2E) (Fig. 6 A). These mutations ablate binding of MUNC13 to liposomes in vitro and severely affect evoked postsynaptic currents and SV docking (Quade et al., 2019; Padmanarayana et al., 2021). MUNC13 C2C 2E localized to presynaptic regions, as shown by co-localization of presynaptic marker VGLUT1 (Fig. 6 B; and Fig. S8, C and F). In MUNC13 DKO cells, DCV exocytosis was not rescued with MUNC13-1 C2C 2E (Fig. 6, C and D), while DCV pool sizes were unaffected (Fig. S8, J–L). These results show that, in line with results previously shown for SV exocytosis, the MUNC13 C2C domain is essential for DCV exocytosis.

## Discussion

**Figure 7.**
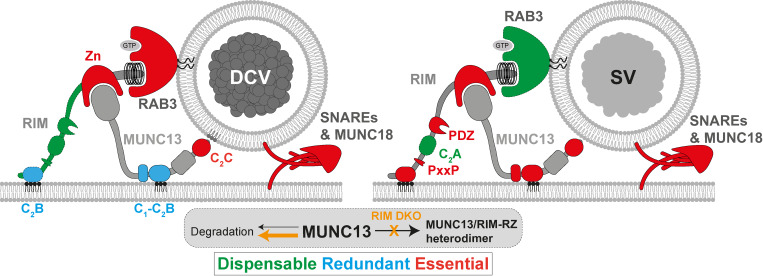
**Essential components of exocytosis working model compare the essential components for DCV exocytosis (left) and SV exocytosis (right) discussed in this study.** RAB3 binds to the RIM N terminus and is essential for DCV exocytosis but dispensable for SV exocytosis. RIM also stabilizes and activates MUNC13 through its N terminus. The whole RIM C terminus, including the PDZ, C2A, PxxP, and C2B domains, is dispensable for DCV exocytosis, while the PDZ, PxxP, and C2B domains are essential for Ca^2+^ channel coupling and SV exocytosis. MUNC13 is essential for DCV and SV exocytosis, and its C2C domain is essential. RIM C2B domain and MUNC13 C1-C2B polybasic face are redundant for DCV exocytosis (light blue) while both are crucial for SV exocytosis.

In this study, we show that MUNC13 is essential for DCV exocytosis, while RIM is crucial for maintaining MUNC13 levels. DCV exocytosis was ablated in MUNC13 DKO neurons and was not restored by expression of RIM1. Conclusions obtained with our validated DCV-reporter, NPY-pHluorin, were confirmed with mass spectrometry analysis of endogenous neuropeptide release. MUNC13 protein levels were severely diminished in RIM DKO neurons and were rescued by inhibiting protein degradation or re-expressing RIM N terminus. Proteasome inhibition also increased DCV exocytosis in RIM DKO neurons to some extent. While mutating either plasma membrane–binding domain of RIM or MUNC13 did not affect DCV exocytosis, mutating both or MUNC13s C2C vesicle membrane–binding domain fully abolished DCV exocytosis. We conclude that MUNC13, and in particular its C2C domain, is essential for DCV exocytosis, that at least one functional membrane-binding domain of either RIM or MUNC13 is needed, and that RIM prevents MUNC13 degradation, thereby enabling DCV exocytosis (Fig. 7).

### MUNC13 is required for DCV exocytosis

No DCV exocytosis was observed in the absence of MUNC13 (Figs. 1, 2, and 3; and Fig. S4, A–H). Loss of MUNC13-2 did not affect DCV exocytosis when compared with WT neurons (Fig. S1, A–C), suggesting that MUNC13-1 is the predominant isoform controlling DCV exocytosis. Using network cultures, MUNC13 was previously shown to result in a ∼60% decrease in DCV exocytosis, using semaphorin-3a–pHluorin to visualize DCV exocytosis (van de Bospoort et al., 2012). In this study, using NPY-pHluorin as a reporter for DCV exocytosis, we observed a complete loss of DCV exocytosis, in line with the phenotype in autaptic neurons (Fig. 2 and Fig. S4, A–H). This conclusion was confirmed by the loss of the release of endogenous neuropeptides (Fig. 3). This discrepancy with our previous study may be explained by the fact that semaphorin-3a does not fully co-localize with endogenous DCV markers (de Wit et al., 2006). Thus, it is plausible that the remaining DCV exocytosis previously observed in the MUNC13 DKO is a result of mis-localization of semaphorin-3a to different compartments. MUNC13 DKO neurons present one of the most extreme phenotypes in the context of SV exocytosis, showing no spontaneous or evoked release of SVs and impaired vesicle docking (Camacho et al., 2021; Deng et al., 2011; He et al., 2017; Kalyana Sundaram et al., 2021; Magdziarek et al., 2020; Quade et al., 2019; Tan et al., 2022; Wang et al., 2019; Varoqueaux et al., 2002). Hence, the essential role of MUNC13 is conserved across the two main regulated secretory pathways in neurons.

### MUNC13 protein levels depend on RIM

MUNC13 levels drop dramatically upon the loss of RIM and are rescued by expressing the RIM N terminus and full-length RIM1 (Fig. 4, A–D, Fig. 6, and Fig. S6, A–E). Furthermore, MUNC13 levels are rescued in the RIM DKO by adding the proteasome inhibitor MG132 or autophagy inhibitor SAR405 (Fig. 4, E–G; Fig. S6, F and G; and Fig. S7, A–C). Interestingly, RIM levels are also increased upon addition of MG132 in control neurons, which most likely also contributes to the increased MUNC13 levels (Fig. S6, F and G). In line with this observation, when RIM1 is overexpressed in control cells, MUNC13 protein levels are also increased (Fig. 4, E–G). Conversely, RIM protein levels are not significantly affected by loss of MUNC13 (Fig. S6, F and G). These observations suggest that MUNC13 levels are regulated by RIM by protecting it from degradation by the proteasome. Previous studies showed that the RIM zinc finger domain performs this task by binding the MUNC13 C2A domain to form a heterodimer, thereby inhibiting MUNC13 homodimer formation (Camacho et al., 2017; Deng et al., 2011; Lu et al., 2006). Taken together, we conclude that the RIM N terminus is a key regulator of MUNC13 levels, whereas RIM levels do not depend on MUNC13.

### RIM and MUNC13 synergistically control DCV exocytosis

In MUNC13 DKO (and QKO) neurons, the loss of DCV exocytosis is not rescued by re-expressing RIM1 (Fig. 2, Fig. S3, G–I, and Fig. S4, I–K), while, conversely, DCV exocytosis is restored in QKO neurons by re-expressing MUNC13-1 (Fig. S4, L–N), similar to previous studies using RIM DKO cells (Persoon et al., 2019). Moreover, MUNC13 levels are strongly reduced in the RIM DKO, while RIM levels are unaffected in MUNC13 DKO neurons, as discussed above (Fig. 4 and Fig. S6, A–E). DCV exocytosis in RIM DKO cells is rescued by a small N-terminal fragment of RIM, containing RAB3 and MUNC13-binding sites (Fig. 1), but not with MUNC13-binding–deficient RIM (Persoon et al., 2019). Altogether, these observations suggest that MUNC13 is crucial for DCV exocytosis and that RIM regulates its availability. The RIM–MUNC13 interaction also activates MUNC13’s functionality, allowing it to bind to other interacting partners such as syntaxin-1. In the absence of RIM, SV priming is only rescued by expressing a constitutively monomeric MUNC13 mutant, which is unable to form homodimers (Deng et al., 2011). The fact that WT MUNC13, which theoretically forms homodimers in the absence of RIM, rescues DCV exocytosis in RIM DKO neurons (Persoon et al., 2019), suggests that the level of MUNC13, but not necessarily its activation, is critical for DCV exocytosis. In line with this observation, we showed that rescuing MUNC13 levels in RIM DKO neurons, which is also theoretically in an inactive homodimer conformation, also increased DCV exocytosis to some degree (Fig. 4 and Fig. S7, D–H). While the MUNC13 C2A domain was demonstrated to form a stable homodimer in vitro, to the best of our knowledge the full-length MUNC13 homodimer has not been shown. It is possible that when overexpressed or rescued through protein degradation inhibition, some MUNC13 is in a monomeric conformation sufficient to drive DCV but not SV exocytosis. Alternatively, another pathway or protein may functionalize MUNC13 for DCV exocytosis. Inhibiting the proteasome may also increase the levels of other synaptic proteins, which could contribute to the observed effect. Nevertheless, the fact that DCV exocytosis is possible in RIM DKO neurons when MUNC13 levels are increased, either by overexpression or proteasome inhibition, is striking and not observed for SV exocytosis. The particularly strong effect on DCV exocytosis in RIM DKO neurons may be explained by the fact that loss of RIM also results in diminished MUNC13 protein levels. However, a RAB3-binding–deficient RIM1 mutant does not fully rescue DCV exocytosis in RIM DKO neurons, suggesting an independent, essential function for RIM as well (Persoon et al., 2019). Previous work has also implicated RIM in Ca^2+^ channel localization, dopamine release, and SV localization and priming (Zarebidaki et al., 2020; Robinson et al., 2019; Han et al., 2015; Kaeser et al., 2011; Schoch et al., 2002). In light of our results, these phenotypes could be explained by the fact that MUNC13 protein levels are diminished in neurons lacking RIM or that RIM is not available to activate MUNC13.

### RIM and MUNC13 membrane interactions control DCV exocytosis in a redundant manner

Mutating either the RIM or MUNC13 membrane–binding domain did not significantly affect DCV exocytosis (Fig. 5, E and F; and Fig. S8, G–I). However, when both domains are mutated, DCV exocytosis is not rescued (Fig. 5, E and F). These observations suggest that DCV exocytosis requires at least one link to the membrane from either RIM or MUNC13. Interestingly, in contrast to DCV exocytosis, SV exocytosis is strongly reduced when either the RIM– or MUNC13–membrane interaction is disrupted (Camacho et al., 2021; de Jong et al., 2018). These data indicate that for DCV exocytosis, the RIM C2B and MUNC13 C1-C2B polybasic face have a redundant function in membrane binding, while for SV exocytosis these domains are both essential. A functional link to the membrane, and in particular to PIP_2_-rich regions, boosts Ca^2+^ entry and also increases the affinity of fusion machinery to Ca^2+^ (Suh et al., 2010; Walter et al., 2017). SV exocytosis must be extremely rapid and synchronous to allow for efficient information transfer, while DCV exocytosis displays slower kinetics and requires a prolonged stimulation (Persoon et al., 2018; Baginska et al., 2023; Bartfai et al., 1988). This difference in required spatiotemporal precision could explain why only one link with the membrane is sufficient for DCV, but not SV exocytosis.

### Vesicle membrane interactions are essential for DCV exocytosis

DCV exocytosis is abolished when the MUNC13 C2C domain is mutated (Fig. 6, C and D; and Fig. S8, J–L). The MUNC13 C2C domain binds weakly to liposomes in a calcium-independent manner, suggesting that the MUNC13 C2C domain stabilizes vesicles at fusion sites by interacting with lipids or proteins in the vesicle membrane (Quade et al., 2019; Padmanarayana et al., 2021). SV exocytosis is also significantly affected by mutating the MUNC13 C2C domain; however, the effect is less extreme compared with DCV exocytosis (Quade et al., 2019; Padmanarayana et al., 2021). This is possibly due to the fact that SVs are more tightly clustered at the active zone and may need less stabilization at the plasma membrane by MUNC13. For DCVs, it is unknown whether MUNC13 binds to vesicles at the release site or earlier in the DCV lifetime. Previously, it was found that the RIM N terminus co-traffics with DCVs in neurons, presumably through its interaction with RAB3 (Persoon et al., 2019). It is possible that MUNC13 also co-travels with DCVs through its C2C domain and its interaction with RIM. This is consistent with the observation that DCVs travel throughout axons and dendrites and can fuse outside synapses (Persoon et al., 2018). All previous and current data considered, it is feasible that MUNC13 stabilizes DCVs at their release sites by forming a link between the vesicle and plasma membrane through its C2C and C1-C2B domains. In addition to the possible MUNC13 C2C–DCV interaction, VAMP2/synaptobrevin2 (VAMP2/syb2) and RAB3 are most likely stable DCV membrane proteins and are both essential for DCV exocytosis (Persoon et al., 2019; Hoogstraaten et al., 2020). VAMP2/syb2 is part of the SNARE complex that drives membrane fusion, and RAB3 interacts with RIM, which also provides a link to the plasma membrane (Schoch et al., 2001; Persoon et al., 2019). Overall, it is clear that the MUNC13 C2C, SNARE, and RAB3–RIM interactions at the DCV membrane are nonredundant for DCV exocytosis. While it is possible that all three interactions are needed in parallel to stabilize a DCV at the target membrane, the different interactions may also be crucial for different steps in the secretory pathway.

### Conservation of mechanisms between DCV and SV exocytosis

The core machinery controlling exocytosis is largely conserved between SV and DCV secretory pathways. DCV and SV exocytosis both depend on SNARE complex formation of the same three proteins (VAMP2/syb2, SNAP25, and syntaxin-1), and this study shows that MUNC13 function is also conserved (Figs. 2 and 7) (Arora et al., 2017; Hoogstraaten et al., 2020; Puntman et al., 2021; Schoch et al., 2001). However, some mechanistic differences between the two pathways are also emerging. RAB3A is essential for DCV exocytosis but largely dispensable for SV exocytosis (Fig. 7) (Geppert et al., 1994; Persoon et al., 2019; Schlüter et al., 2004, 2006). Perturbing the RAB3–RIM interaction also has a much stronger effect on DCV exocytosis when compared with SV exocytosis (Deng et al., 2011; Persoon et al., 2019). RAB3A-dependent aspects of exocytosis may have become redundant with other regulatory proteins specific for SV exocytosis. On the other hand, previous data and the current study show that a fragment of the RIM N terminus is sufficient to drive DCV exocytosis, while the other domains of RIM are needed for SV exocytosis (Figs. 1, 5, and 7) (Persoon et al., 2019; Deng et al., 2011; de Jong et al., 2018). The observation that WT MUNC13 is sufficient to support DCV, but not SV exocytosis (even when overexpressed) in the absence of RIM, is a further indication that RIMs function in the context of DCV exocytosis is more limited to sustaining MUNC13 levels, while in the context of SV exocytosis it is also crucial for organizing the active zone and localization of SVs to Ca^2+^ channels. Interestingly, in the early life-forms Porifera (sponges) and Placozoa, the RIM ortholog lacks the PDZ domain that is essential for coupling to Ca^2+^ channels and vital for SV exocytosis. These ancient species contain neuropeptides and their receptors but lack ultrafast synaptic transmission (Piekut et al., 2020). Our results also show that the membrane-binding domains of RIM and MUNC13 are redundant for DCV exocytosis, while both are crucial for SV exocytosis (Fig. 5) (Camacho et al., 2021; de Jong et al., 2018). The different redundancies that the two pathways developed may help to explain the differences in release kinetics, effective stimulation, and localization that evolved between DCVs and SVs. The C terminus of RIM provides a link to Ca^2+^ channels and PIP_2_-rich patches in the plasma membrane, ensuring a tight coupling of SVs to release sites to allow for rapid fusion. The fact that the C2B domains of RIM and C1-C2B polybasic face of MUNC13 are redundant for DCV, but not SV exocytosis, is further evidence that tight coupling to the (PIP_2_-rich) membrane is crucial for fast neurotransmission. The fact that DCVs fuse in and outside of synapses could begin to explain the strong dependency on RAB3; RAB3 has been shown to couple traveling cargo to motor proteins via the protein MADD and ARL8A/B, allowing for transport throughout the neurites (Hummel and Hoogenraad, 2021). Additionally, other RAB proteins may be present in active zones that make RAB3 redundant for SV exocytosis, such as RAB27 (Takamori et al., 2006). All in all, the exocytic principles for the core fusion machinery are largely conserved between SVs and DCVs, but this study and other recent evidence are beginning to identify unique redundancies that help us understand how the two secretory pathways evolved and diverged.

## Materials and methods

### Animals

*Rim1/2*; *Munc13-1/2* quadrupole conditional null mice were generated by breeding the *Rim1*^*lox/lox*^*/Rim2*^*lox/lox*^ conditional null line (Kaeser et al., 2011) with *Munc13-1*^*hz*^*/Munc13-2*^*ko/ko*^ double null mice (Varoqueaux et al., 2002). As *Munc131/2* double null confers lethality, E18 pups were obtained by caesarean section of pregnant females from timed matings of *Rim1*^*lox/lox*^*Rim2*^*lox/lox*^; *Munc13-1*^*hz*^*Munc13-2*^*ko/ko*^ mice and genotyped by PCR as described previously (Meijer et al., 2012). For the preparation of glia, newborn Wistar rats were used. All animal experiments were approved by the “Centrale Commissie Dierproeven” (Central Commission for Animal Experiments) of the Netherlands Government and were performed according to the Netherlands Law on Animal Research (Wet op Dierproeven) in full agreement with the Directive 2010/63/EU with local approval by and under supervision of the Animal Welfare Body VU and VU Medical Center (VUmc).

### Neuronal cultures

Mouse hippocampal and cortical cultures were prepared from E18 pups. Hippocampi and cortices were dissected in HBSS (H9394; Sigma-Aldrich) supplemented with 10 mM HEPES (pH 7.4, 15630056; Gibco). After dissection, tissue was digested in 0.25% trypsin (15090046; Gibco) at 37°C for 15 min. After tryptic digestion, the tissue was washed twice with HBSS and triturated with a fire-polished Pasteur pipette in DMEM (VWRC392–0415; VWR Life Science) supplemented with 10% FCS (10270; Gibco), nonessential amino acids (M7145; Sigma-Aldrich), and antibiotics (penicillin/streptomycin, 15140122; Gibco). After trituration, the cell suspension was spun down and resuspended in Neurobasal medium (21103049; Gibco) supplemented with B27 (17504044; Gibco), 10 mM HEPES, GlutaMAX (35050038; Gibco), and antibiotics (penicillin/streptomycin, 15140122; Gibco). To obtain single neuron cultures for live-cell imaging and immunocytochemistry experiments, neurons were plated on rat glial micro-islands on glass coverslips at a density of 2,000 cells/well. Micro-islands were generated as described previously (Meijer et al., 2012) by plating rat glia on micro-islands of growth permissive substrate (mix of collagen I and poly-D-lysine, 354236; Corning and P6407; Sigma-Aldrich, respectively) that were printed on a layer of 0.15% agarose. For mass culture experiments, neurons were plated on glass coverslips covered with rat glia at a density of 30 × 10^3^ cells/well. For western blot, neurons were plated on plastic 6-well plates coated with 0.01% poly-L-ornithine (P4957; Sigma-Aldrich) and 2.5 μg/ml laminin (L2020; Sigma-Aldrich), diluted in Dulbecco’s PBS (14190-250; Gibco) at a density of 350 × 10^3^ cells/well. All cultures were maintained in a humidified incubator at 37°C and 5% CO_2_ for 14–16 days.

### Lentiviral constructs and infection

To induce loss of RIM, cultures were infected at the time of plating or at DIV1 with a Cre-recombinase lentivirus under the control of a synapsin promoter (pSyn-Cre-mCherry or pSyn-Cre-GFP). Control neurons were infected with inactive Cre-recombinase (ΔCre). For rescue experiments, neurons were infected at the time of plating or at DIV1 with a lentivirus encoding the rescue construct. The RIM rescue constructs RIM-FL, RIM-RZ, and RIM C2B 2CE were described previously (Deng et al., 2011; de Jong et al., 2018; Persoon et al., 2019). They were generated from a rat RIM1 expression plasmid and contained an HA-tag. The RIM-RZ construct also contained an mScarlet tag on the C terminus. The MUNC13-1 rescue constructs were described previously (Quade et al., 2019; Camacho et al., 2021) and contained EGFP before the N terminus with a nuclear localization sequence of nucleoplasmin to ensure nuclear localization of EGFP and a FLAG-tag on the C terminus. NPYsd-pHluorin was infected on DIV9–10. The construct was generated by removing the CPON sequence in NPY, and mutating the last two amino acids that constitute the GKR receptor–binding site. All constructs were cloned into synapsin-driven constructs, sequence verified, and subcloned into pLenti vectors (Table 1).

**Table 1.** List of constructs used in this study

Name	Source
pSyn(pr)-Cre-mCherry, pSyn(pr)-ΔCre-mCherry	Wolzak et al. (2022)
pFSW-ncl-Cre, pFSW-ncl-ΔCre	Kaeser et al. (2011)
pSyn(pr)-RIM1-HA, pSyn(pr)-RIM1-RZ-HA	Deng et al. (2011), Kaeser et al. (2011), Persoon et al. (2019)
pSyn(pr)-RIM1-C2Bmut-HA	de Jong et al. (2018)
pSyn(pr)-MUNC13-1-Flag, pSyn(pr)-MUNC13-1(K603E)-Flag, pSyn(pr)-MUNC13-1(R1598, F1658E)-Flag	Quade et al. (2019), Camacho et al. (2021)
pSyn(pr)-NPYsd-pHluorin	Subkhangulova et al. (2023), Nassal et al. (2022)

### Immunocytochemistry

Hippocampal cultures were fixed in 3.7% formaldehyde (15680; Electron Microscopies Sciences) in PBS (pH 7.4) for 20 min at RT. Cells were washed three times in PBS and permeabilized for 5 min with 0.5% Triton X-100 (T/3751/08; Fisher Chemical) in PBS. Cells were incubated for 30 min in 2% normal goat serum (16210-072; Gibco) and 0.1% Triton X-100 in PBS to block nonspecific binding of antibodies. Incubation with primary antibodies were performed for 2 h at RT or overnight at 4°C. Incubation with secondary antibodies was performed for 1 h at RT. See details of primary antibodies in Table 2. Alexa Fluor–conjugated secondary antibodies were from Invitrogen (1:500). After incubation with primary and secondary antibodies, coverslips were washed in PBS and mounted with Mowiol 4–88 (81381; Sigma-Aldrich) and imaged on a confocal A1R microscope (Nikon) with LU4A laser unit using a 40× oil immersion objective (NA = 1.3). Images were acquired at 1,024 × 1,024 pixels as z-stacks (5 steps of 0.25 µm). Maximum projection images were used for analysis. Confocal imaging settings were kept constant for all images within an experiment.

**Table 2.** List of primary antibodies used

Name	Type	Dilution	Company
MAP2	Polyclonal	1:500 (ICC)	5392; Abcam
VGLUT1	Polyclonal	1:1,000 (ICC)	135302; SySy
HA	Monoclonal	1:500 (ICC)	12CA5; Roche
FLAG	Monoclonal	1:1,000 (ICC)	F1804; Sigma-Aldrich
RIM1	Polyclonal	1:500 (ICC), 1:1,000 (WB)	140003; SySy
MUNC13-1	Polyclonal	1:1,000 (ICC, WB)	126103; SySy
α-tubulin	Monoclonal	1:10,000 (WB)	302211; SySy
p62/SQSTM1	Polyclonal	1:2,000 (WB)	PA5–20839; Thermo Fisher Scientific

### Secretome analysis by mass spectrometry

#### Sample preparation

DIV21 hippocampal neurons grown in high-density culture (30 × 10^3^ cells/well in a 12-well plate) were washed four times with pre-warmed normal Tyrode’s buffer (for recipe see section Live-cell imaging) for 3 min. After washing, cells were incubated with 250 μl pre-warmed normal Tyrode’s buffer for 5 min 240 μl medium was collected and immediately put on ice (baseline). Cells were stimulated with 250 μl high potassium Tyrode’s (60 mM KCl), after which 240 μl medium was collected and placed on ice (stimulation). Baseline and stimulation samples were spun down for 10 min at 4°C at 1,000 *g*. 220 μl supernatant was transferred to a new tube and spun down for 10 min at 4°C at 18,000 *g*. 210 μl supernatant was transferred to a new tube and immediately stored until use at −80°C. All tubes used were Eppendorf protein lo-bind tubes (cat. no. EP0030108116-100EA).

#### LC-MS analysis

Each sample of supernatant was acidified up to 0.5% formic acid, and equal volumes (100 μl) were loaded onto an Evotip Pure (Evosep). Peptide samples were separated by standardized 30 samples per day method on the Evosep One liquid chromatography system, using a 15 cm × 150-μm reverse-phase column packed with 1.5-µm C18-beads (EV1137 from Evosep) connected to a 20-µm ID ZDV emitter (Bruker Daltonics).

Peptides were electro-sprayed into the timsTOF HT mass spectrometer (Bruker Daltonics) equipped with CaptiveSpray source and measured with the following settings: scan range 100–1,700 m/z, ion mobility 0.65–1.6 Vs/cm^2^, ramp time 200 ms, accumulation time 200 ms, and collision energy decreasing linearly with inverse ion mobility from 59 eV at 1.6 Vs/cm^2^ to 20 eV at 0.6 Vs/cm^2^.

Operating in high-sensitivity dda-PASEF mode, each cycle took 1.03 s and consisted of 1 MS1 full scan followed by 4 PASEF scans per top-N acquisition. The intensity threshold for precursor selection was set to 2,500 a.u. with a target intensity of 20,000 a.u. Reaching the target intensity resulted in active exclusion for 0.4 min, with additional reconsideration upon a further fourfold increase in intensity. The quadrupole isolation width was set to 2 m/z for precursors below 700 m/z, 3 m/z for precursors above 800 m/z, and interpolated linearly between 700 and 800 m/z. The precursor region filter was disabled, and precursors were selected with a charge between 0 and 5. Ion mobility was auto calibrated at the start of each sample (calibrant m/z, 1/K0: 622.029, 0.992 Vs/cm^2^; 922.010, 1.199 Vs/cm^2^; 1,221.991, 1.393 Vs/cm^2^).

SCG-1 detection (Fig. S5) was performed using a proteomics pipeline previously described in Thanou et al. (2023).

#### Data analysis

dda-PASEF raw data were processed with Fragpipe v22.0 (Kong et al., 2017), using the nonspecific peptidome workflow. A custom FASTA sequence database was created that contains neuropeptides obtained from NeuroPep (downloaded on 2023-08–29) (Wang et al., 2024) and peptide sequences annotated in the UniProt mouse proteome (downloaded on 2023-08–20) with a length between 6 and 65 amino acids. Fixed modification of cysteine was disabled. MS1 quantification was enabled, with MBR disabled. All other settings were left as default.

Peptide intensity values of all detected neuropeptides were summed per sample and plotted as-is in Fig. 3 B. The log_10_ intensity value of each individual neuropeptide is shown in Fig. 3 C.

For SCG-1 peptides detected using a proteomics pipeline, SCG-1 peptide intensities were averaged across replicates and normalized to baseline control.

### SDS-PAGE protein quantitation analysis

To study the effects of the proteasome inhibitor MG132 on RIM and MUNC13 protein levels, 10 mM MG132 (Calbiochem) was added to high-density cortical cultures on DIV14–16. After 6 h incubation with MG132, the cells were washed with PBS and lysed in Laemmli sample buffer (2% SDS [M107; VWR Chemicals], 10% glycerol [818709; Merck], 50 mM DTT [11896744; Thermo Fisher Scientific], 60 mM Tris-HCl [37180; Serva], pH 6.8, and 0.01% Bromophenol blue [A3640; Applichem]). To study the effects of the autophagy inhibitor SAR405 on MUNC13-1 levels, 5 μM SAR405 (5330630001; Merck) was added to high-density cortical cultures on DIV16. After 24 h incubation, cell lysates were collected in the same way as stated above. Cell lysates were heated at 95°C for 5 min, separated on homemade Tris-glycine 8% SDS-polyacrylamide gels, and transferred to nitrocellulose membranes (1620115; Bio-Rad) using a wet tank transfer method. Membranes were blocked for 20 min at RT in 5% milk powder (115360500; Sigma-Aldrich) dissolved in PBS containing 0.1% Tween20 (8.22184.0500; VWR). Membranes were incubated in primary antibodies diluted in 5% milk PBS containing 0.1% Tween20 overnight at 4°C. Primary antibodies with dilutions are listed in Table 2. Secondary antibodies coupled to horseradish peroxidase were applied at 1:10,000 dilution for 1–2 h at RT. Detection was performed using SuperSignal West Femto Maximum Sensitivity Substrate (34095; Thermo Fisher Scientific) on the Odyssey Fc imaging system (LI-COR Bioscience). Analysis of signal intensities was performed using Image Studio Lite Software, and all signal intensities were normalized to the intensity of a loading control (α-tubulin).

### Live-cell imaging

Live-imaging experiments were performed on a Nikon Ti-E Eclipse inverted microscope system (Camera: Photometrics Prime 95B, objective lens: oil immersion, 60×, NA = 1.4). NIS elements software (AR 5.30.06) controlled the microscope and image acquisition. Coverslips were placed in an imaging chamber and continually perfused with Tyrode’s solution (119 mM NaCl, 2.5 mM KCl, 2 mM CaCl_2_*2H_2_0, 2 mM MgCl_2_*6H_2_0, 25 mM HEPES and 30 mM Glucose*H_2_0, pH 7.4, and Osmol 280). Time-lapse imaging (2 Hz, GFP filter, and 200-ms exposure time) consisted of 30-s baseline recording followed by 16 trains of 50 action potentials at 50 Hz with a 0.5-s interval between trains. The field stimulation was applied by parallel platinum electrodes powered by a stimulus isolator (A385; WPI), regulated by a Master-9 pulse generator (A.M.P.I.). To visualize the remaining pool of DCVs after stimulation, the intracellular pH was neutralized by application of Tyrode’s solution containing 50 mM NH_4_Cl through glass capillary barrels. Calcium influxes were recorded by incubating the cells with 1 μM Fluo-5F-AM (F14222; Molecular Probes, dissolved in DMSO) for 10 min prior to imaging and stimulation. All live-cell imaging experiments were performed at RT, and a maximum of 5 islands per coverslip were recorded.

### Imaging analysis

#### ICC analysis

The staining intensity of the maximum projection images was measured using SynD (Schmitz et al., 2011) and a custom-made script in Fiji. Neurite masks were generated in SynD using Map2 signal, and used in Fiji to measure the neurite intensity of probes. Co-localization analysis was performed by plotting the profile intensities of two channels along a neurite.

#### DCV exocytosis

Analysis of time-lapse recordings was performed as previously described (Moro et al., 2021). In short, DCV exocytosis events were detected by a sudden rise in fluorescence intensity. 3 × 3 pixel regions of interest (ROIs) were placed using a semiautomated script in Fiji (Moro et al., 2021). Missed fusion events were placed manually. The fluorescence intensity trace per ROI was measured and loaded into a custom-made MATLAB script. Fusion events were counted when ΔF/F_0_ > 2*SD and rise time <1 s. The soma was excluded from analysis, as some datasets contained a (Δ)cre-EGFP construct that localized to the nucleus, thereby masking the somatic fusion events. The remaining pool size of DCVs (here, soma was excluded as well) was determined as the number of fluorescent puncta during application of 50 mM NH_4_Cl. The average intensity all ROIs was calculated and used to represent the intensity of one single vesicle. All ROI intensities were normalized to the average intensity, thereby correcting the total pool size for overlapping puncta. The released fraction was calculated by dividing the total amount of fusion events by the remaining pool size. For visualization purposes, the log(1p) of the released fraction was taken (1p = value +1).

#### Calcium imaging

Analysis of calcium in- and efflux was performed in Fiji. 3 neurite-located ROIs (segmented line, line width = 10) were measured per neuron. Normalized ΔF/F_0_ traces were used to calculate the area under the curve (performed in GraphPad Prism).

### Quantification and Statistical analysis

The released fraction of DCV exocytosis was calculated by dividing the number of fusion events by the total remaining pool. The released fractions were subsequently visualized with a log scale. To circumvent zero values in the released fractions, +1 was added to all values before taking the log value. Statistical analyses were performed on raw data (before taking log value) in GraphPad Prism 10.1.1. Shapiro–Wilk test was used to assess whether data were normally distributed. Kruskal–Wallis or one-way ANOVA test followed by Dunn’s multiple comparison post hoc tests were used for more than two experimental groups. Mann–Whitney or *t* test was used to assess two experimental groups. Source data (individual datapoints, mean, SEM, SD, *N* and *n*, P values, statistical tests, and used methods) are available upon request.

### Online supplemental material

Our supplementary material contains supplementary figures that contain data supporting the findings in main figures. Fig. S1 shows the validation of MUNC13/RIM mouse line. Fig. S2 shows the RIM1-RZ rescue construct expression, DCV exocytosis kinetics using NPYsd-pHluorin reporter, and extra exocytosis parameters belonging to Fig. 1. Fig. S3 shows the RIM1 rescue construct validation and extra exocytosis parameters belonging to Fig. 2. Fig. S4 shows the MUNC13 DKO in autaptic and network neuronal cultures; MUNC13, but not RIM, rescues DCV exocytosis in QKO neurons. Fig. S5 shows the peptide counts and abundance and proteomics analysis of SCG-1 belonging to Fig. 3. Fig. S6 shows that the RIM expression levels are unaffected in MUNC13 DKO. Fig. S7 shows that the MUNC13-1 levels increase upon inhibition of autophagy and extra exocytosis parameters belonging to Fig. 3. Fig. S8 shows the MUNC13 K603E, RIM1 C2B 2E, and MUNC13 2CE validation and extra exocytosis parameters belonging to Figs. 4 and 5.

## Supplementary Material

SourceData F4 is the source file for Fig. 4.

SourceData FS3 is the source file for Fig. S3.

SourceData FS6 is the source file for Fig. S6.

SourceData FS7 is the source file for Fig. S7.

## Data Availability

Raw data and plasmids used in this study are available from the corresponding authors upon request.
