# Supplementary figures and images for: RIM and MUNC13 membrane–binding domains are essential for neuropeptide secretion
Source: J Cell Biol. 2025 May 12;224(7):e202409196. doi: 10.1083/jcb.202409196 (PMC12077229; doi:10.1083/jcb.202409196)

**Source Data F4 - Uncropped blot showed in Figure 4C/4F**

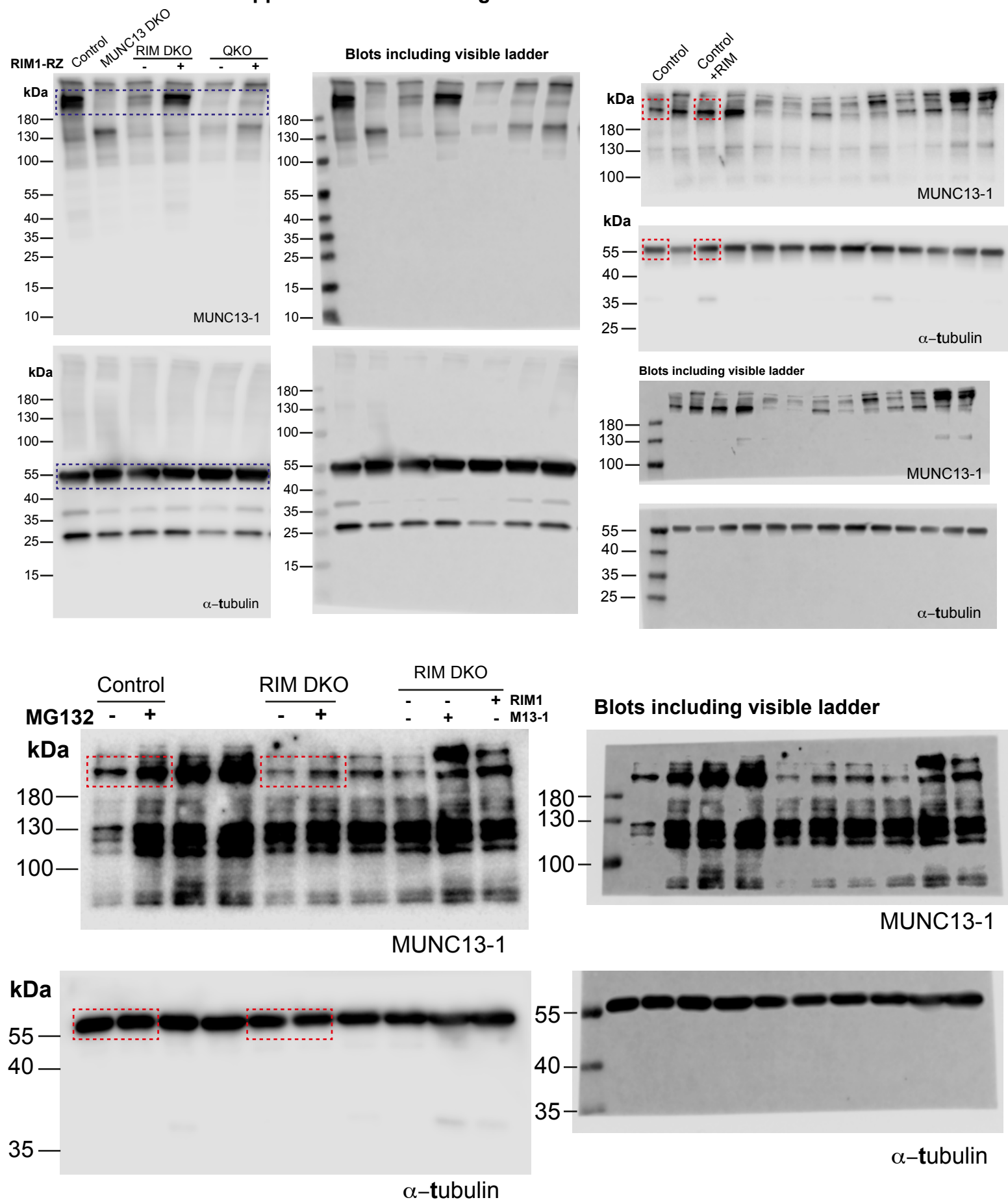

Supplement: SourceData F4 — is the source file for Fig. 4. [file jcb_202409196_sourcedataf4.pdf]

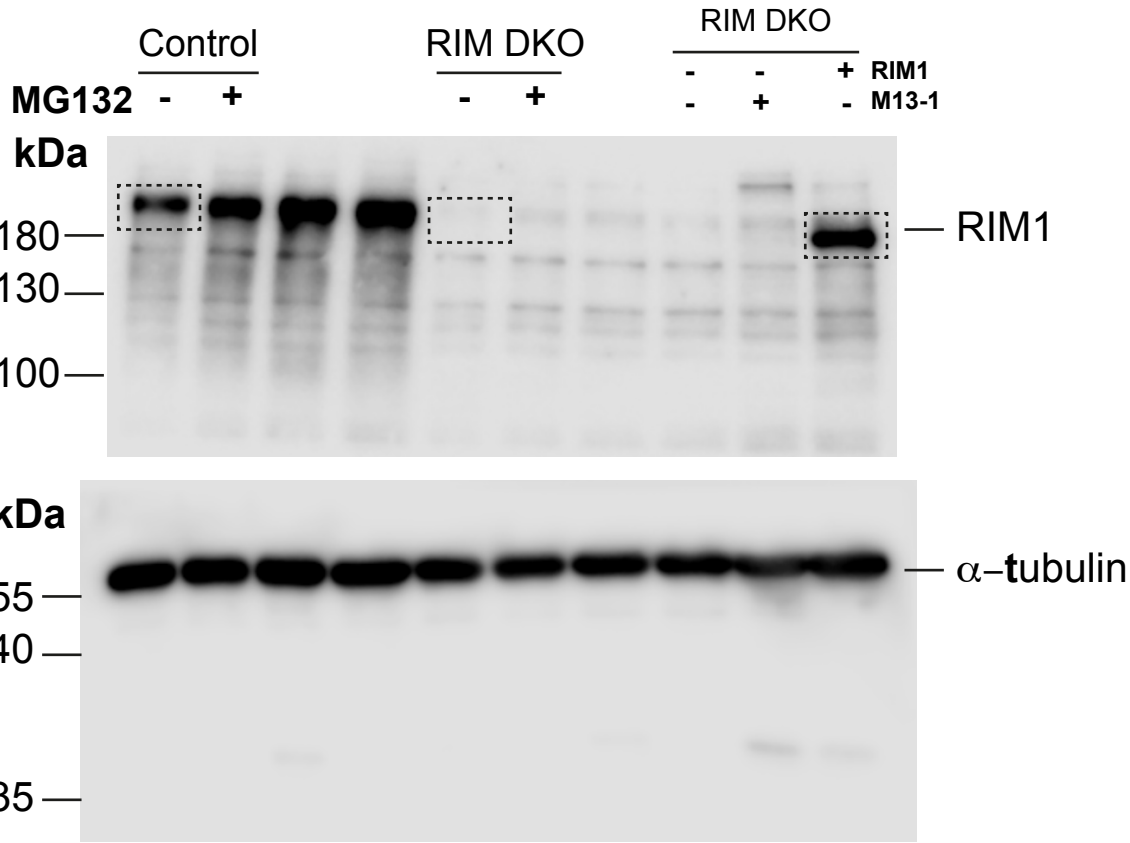

Blots including visible ladder

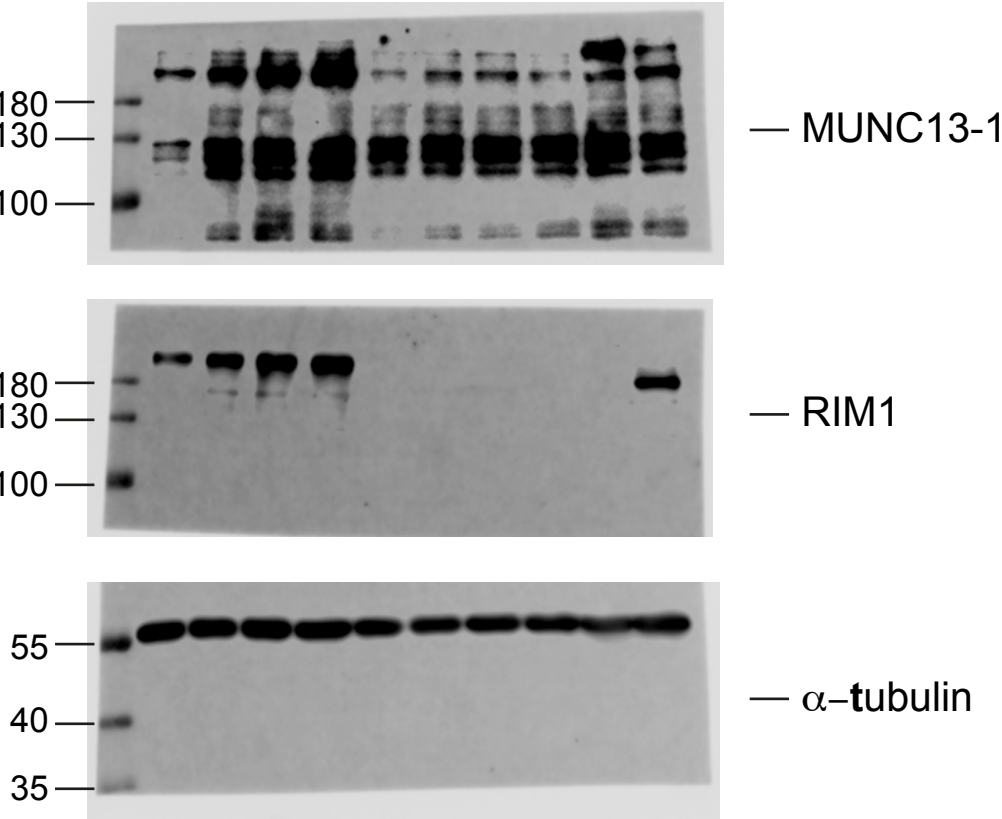

Supplement: SourceData FS3 — is the source file for Fig. S3. [file jcb_202409196_sourcedatafs3.pdf]

Source Data FS6 - Uncropped blot showed in Supplementary Figure S6C/S6F

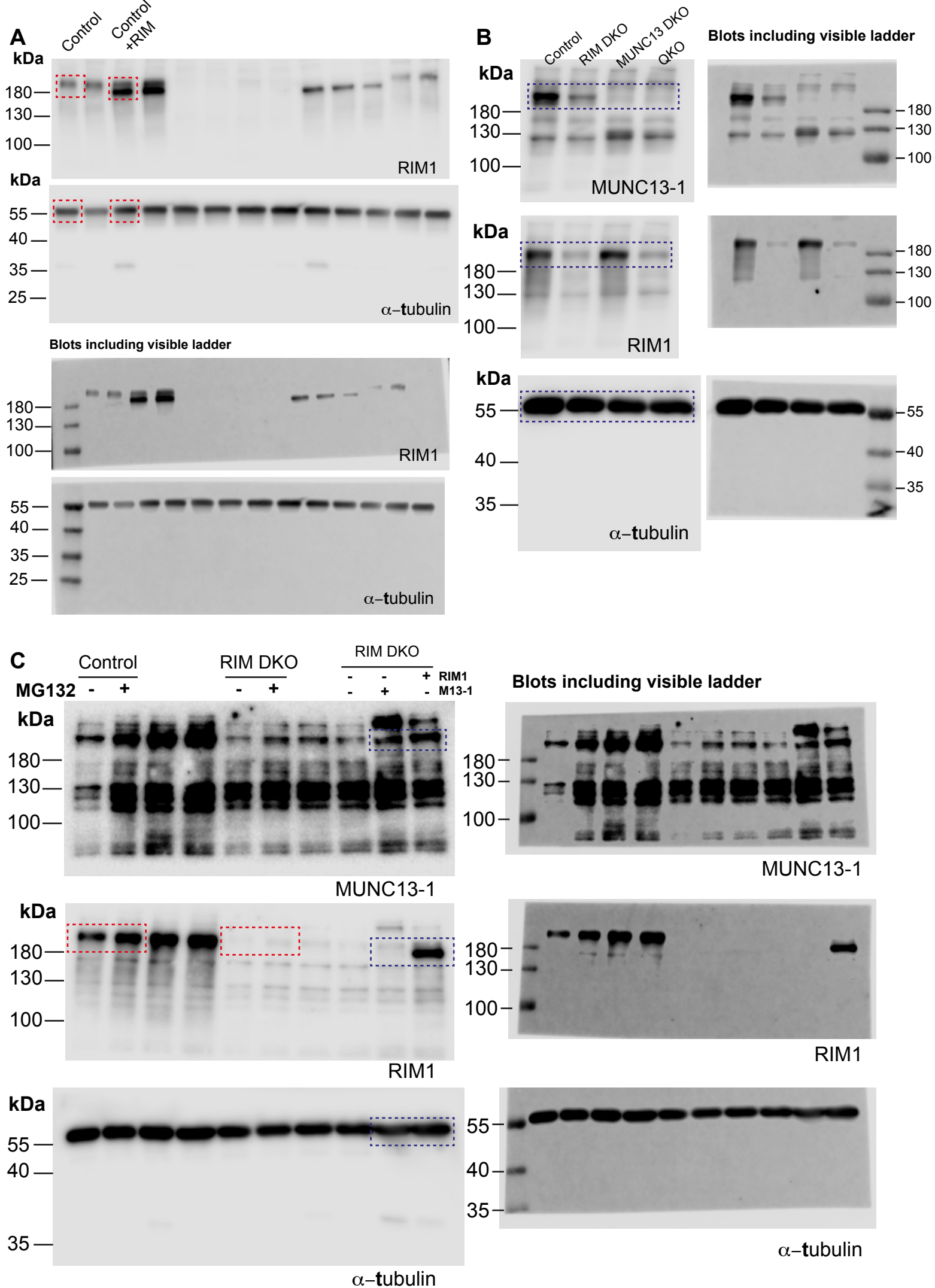

Supplement: SourceData FS6 — is the source file for Fig. S6. [file jcb_202409196_sourcedatafs6.pdf]

Source Data FS7 - Uncropped blot showed in Supplementary Figure S7

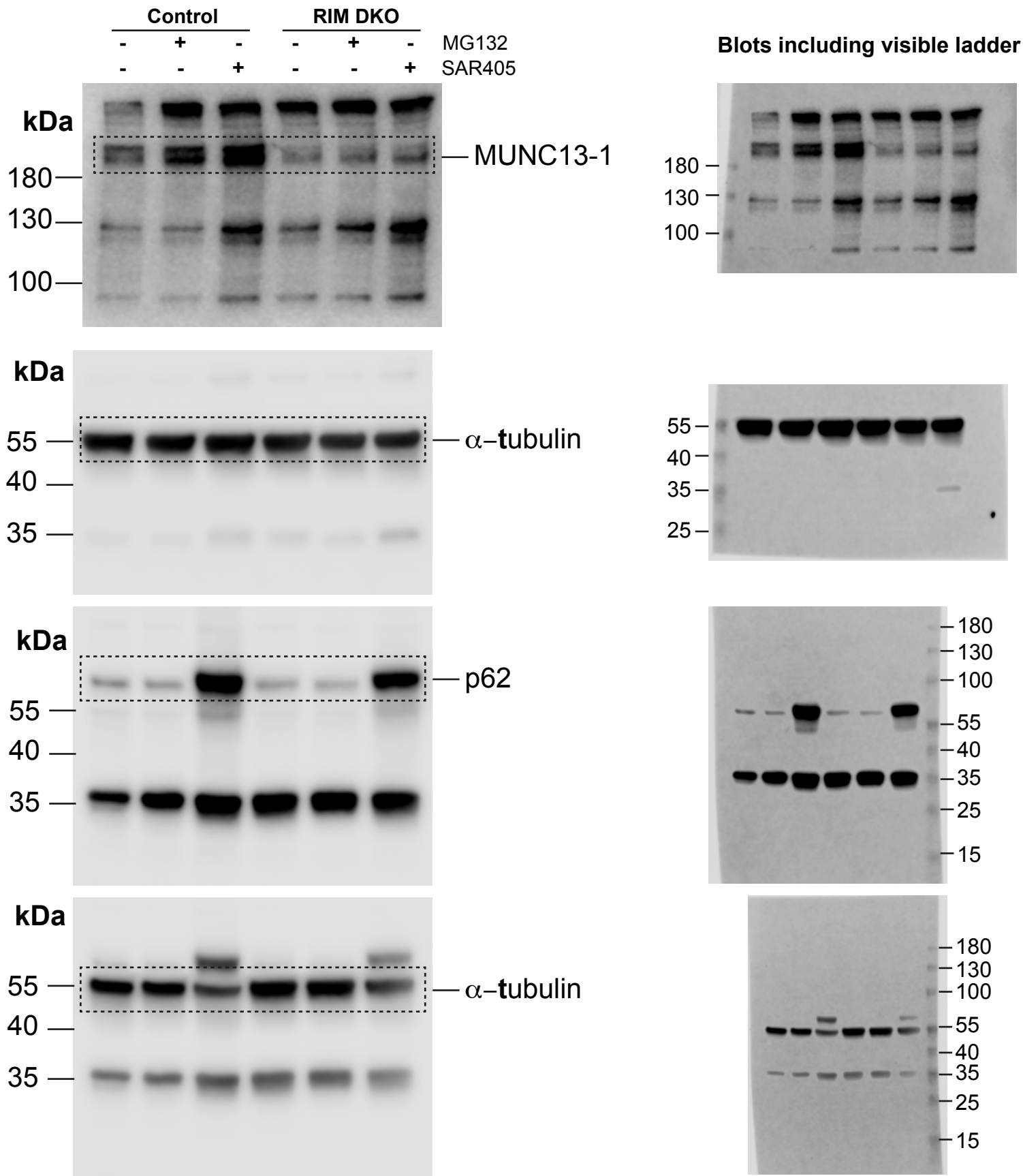

Supplement: SourceData FS7 — is the source file for Fig. S7. [file jcb_202409196_sourcedatafs7.pdf]
